# Characteristics and knowledge synthesis approach for 456 network meta-analyses: a scoping review

**DOI:** 10.1186/s12916-016-0764-6

**Published:** 2017-01-05

**Authors:** Wasifa Zarin, Areti Angeliki Veroniki, Vera Nincic, Afshin Vafaei, Emily Reynen, Sanober S. Motiwala, Jesmin Antony, Shannon M. Sullivan, Patricia Rios, Caitlin Daly, Joycelyne Ewusie, Maria Petropoulou, Adriani Nikolakopoulou, Anna Chaimani, Georgia Salanti, Sharon E. Straus, Andrea C. Tricco

**Affiliations:** 1 Knowledge Translation Program, Li Ka Shing Knowledge Institute, St. Michael’s Hospital, Toronto, Ontario M5B 1W8 Canada; 2 Department of Hygiene and Epidemiology, University of Ioannina, Ioannina, 45110 Greece; 3 Institute of Social and Preventive Medicine, University of Bern, Bern, Switzerland; 4 Institute of Primary Health Care, University of Bern, Bern, Switzerland; 5 Department of Medicine, Faculty of Medicine, University of Toronto, Toronto, Ontario M5S 1A1 Canada; 6 Epidemiology Division, Dalla Lana School of Public Health, University of Toronto, Toronto, Ontario M5T 3M7 Canada

**Keywords:** Mixed-treatment, Multiple treatments, Research reporting, ISPOR, AMSTAR

## Abstract

**Background:**

Network meta-analysis (NMA) has become a popular method to compare more than two treatments. This scoping review aimed to explore the characteristics and methodological quality of knowledge synthesis approaches underlying the NMA process. We also aimed to assess the statistical methods applied using the Analysis subdomain of the ISPOR checklist.

**Methods:**

Comprehensive literature searches were conducted in MEDLINE, PubMed, EMBASE, and Cochrane Database of Systematic Reviews from inception until April 14, 2015. References of relevant reviews were scanned. Eligible studies compared at least four different interventions from randomised controlled trials with an appropriate NMA approach. Two reviewers independently performed study selection and data abstraction of included articles. All discrepancies between reviewers were resolved by a third reviewer. Data analysis involved quantitative (frequencies) and qualitative (content analysis) methods. Quality was evaluated using the AMSTAR tool for the conduct of knowledge synthesis and the ISPOR tool for statistical analysis.

**Results:**

After screening 3538 citations and 877 full-text papers, 456 NMAs were included. These were published between 1997 and 2015, with 95% published after 2006. Most were conducted in Europe (51%) or North America (31%), and approximately one-third reported public sources of funding. Overall, 84% searched two or more electronic databases, 62% searched for grey literature, 58% performed duplicate study selection and data abstraction (independently), and 62% assessed risk of bias. Seventy-eight (17%) NMAs relied on previously conducted systematic reviews to obtain studies for inclusion in their NMA. Based on the AMSTAR tool, almost half of the NMAs incorporated quality appraisal results to formulate conclusions, 36% assessed publication bias, and 16% reported the source of funding. Based on the ISPOR tool, half of the NMAs did not report if an assessment for consistency was conducted or whether they accounted for inconsistency when present. Only 13% reported heterogeneity assumptions for the random-effects model.

**Conclusions:**

The knowledge synthesis methods and analytical process for NMAs are poorly reported and need improvement.

**Electronic supplementary material:**

The online version of this article (doi:10.1186/s12916-016-0764-6) contains supplementary material, which is available to authorized users.

## Background

Remaining up-to-date on healthcare information is a challenge with approximately 75 trials and 11 systematic reviews being published daily [1]. Healthcare professionals and decision-makers increasingly rely on knowledge syntheses, such as systematic reviews and meta-analyses, to keep abreast of the literature and inform decisions based on the totality of evidence [1, 2]. This may explain why systematic reviews and meta-analyses have the highest relative citation impact in health research [3]. However, pairwise meta-analyses are limited by the availability of randomised controlled trials (RCTs) that directly compare one treatment relative to another. This can be particularly problematic when comparing the efficacy of multiple competing interventions, since it is unlikely that RCTs provide direct comparisons for all interventions of interest [4–6].

To overcome this challenge, an extension to pairwise meta-analysis that allows indirect comparisons of multiple competing interventions in the absence of trials involving a direct comparison have been proposed [7, 8]. The indirect method implies that the information available from RCTs of treatment A and treatment B can be compared via a common comparator C (e.g., placebo or usual care) by statistically combining the information from RCTs comparing A versus C and B versus C [4]. When a single model combines information from both direct and indirect comparisons across a network of studies to infer the relative efficacy and safety of multiple interventions, it constitutes a network meta-analysis (NMA). Other terms used for NMA include mixed-treatment comparisons meta-analysis or multiple treatments meta-analysis [9, 10].

The use of NMA has increased rapidly since the mid-2000s [4, 11, 12]. This rapid development has raised concerns about the standardization and transparency of conduct and reporting of NMA publications. Recent publications from the International Society for Pharmacoeconomics and Outcomes Research (ISPOR) [13, 14] and the Preferred Reporting Items for Systematic Reviews and Meta-analyses (PRISMA) extension statement for NMAs [15] have attempted to offer education and guidance on optimal conduct and reporting of NMAs. An overview of reviews exploring the existing publications on quality of reporting in NMAs found several deficiencies [16]. However, an in-depth assessment of the conduct of the knowledge synthesis approaches underlying the NMA is lacking. As such, we aimed to explore the characteristics and methodological quality of knowledge synthesis approaches of NMAs. We also aimed to assess the statistical methods applied using the Analysis subdomain of the ISPOR checklist [17].

## Methods

### Study protocol

A scoping review protocol was developed using the methodological framework proposed by Arksey and O’Malley [18], as well as the methods manual published by the Joanna Briggs Institute Methodology for scoping reviews [19]. The review protocol can be found in Additional file 1: Appendix 1. This scoping review is related to another methodological review that focused on the characteristics and core statistical methodology specific to NMAs in clinical research [20].

### Eligibility criteria

We included NMAs that compared at least four different interventions from RCTs using a valid statistical method for indirect comparisons (e.g., adjusted or anchored indirect comparison method [7, 13]) or NMAs (e.g., hierarchical models). Studies that applied a naïve or invalid indirect comparison approach failing to preserve within-study randomization were excluded [21]. Studies of diagnostic test accuracy and those including animals or only non-randomized studies were also excluded. NMAs in which the number of trials was smaller than the number of interventions were excluded. Both published and unpublished reports in all languages of publication were eligible for inclusion.

### Information sources and literature search

An experienced library technician conducted comprehensive literature searches in MEDLINE, EMBASE, PubMed, and Cochrane Database of Systematic Reviews from inception until April 14, 2015. The MEDLINE search strategy was developed in consultation with the research team and peer-reviewed by an expert librarian using the Peer Review of Electronic Search Strategies (PRESS) checklist [22]. The final search strategy for the MEDLINE database can be found in Additional file 1: Appendix 2. The database search was supplemented by manually searching the references of a relevant systematic review [23] and a pre-existing database of NMAs [11].

### Study selection process

The screening criteria were established a priori and calibrated amongst the team (AAV, AV, SS, PR, MP, AN, AC) with a pilot-test on a random sample of 50 articles. After more than 90% inter-rater agreement was established, pairs of reviewers screened the titles and abstracts independently, and all discrepancies were resolved by a third reviewer (AAV, PR, AC, GS). The same process was followed when screening potentially relevant full-text articles. All levels of screening were performed using our proprietary online tool, Synthesi.SR [24].

### Data items and data abstraction process

A predefined data abstraction form was developed in Excel. The abstracted data included study characteristics (e.g., author, publication year, country of corresponding author, journal name, funding sources) and steps involved in the knowledge synthesis conduct (e.g., protocol use, inclusion criteria, literature search approach, screening and data collection process, quality appraisal). We also collected data on the terminology used to describe NMAs and references of methodology papers that informed the analysis.

The form was calibrated through two pilot-tests amongst the team (WZ, VN, AV, ER, SM, JA, ACT) on a random sample of seven included articles. For this exercise, the team independently abstracted data and a facilitated team meeting was held for feedback and discussion on discrepant items. Upon completion of the pilot-tests, pairs of reviewers (WZ, VN, AV, ER, SM, JA) independently completed data abstraction for the first 215 included articles. The remaining 241 included articles were abstracted by one reviewer and verified by a second reviewer. All discrepancies between reviewers were resolved by a third reviewer (WZ, VN).

### Quality assessment of included NMAs

The quality of the knowledge synthesis methods was appraised using the AMSTAR tool [25]. The AMSTAR tool was created and validated to assess the methodological quality of systematic reviews of RCTs [26]. The tool measures overall quality, where a score of 8 or higher is considered high quality, 4 to 7 is moderate quality, and 0 to 3 is low quality [27]. Information for quality assessment was incorporated into the data extraction form, which was pilot-tested on a random sample of seven included articles that ranged from low to high quality.

To appraise the validity of the analytical methods applied, we used the 6-item Analysis subdomain of the ISPOR checklist for NMAs [17]. To ensure high inter-rater agreement, a workshop on the tool was held with the team and two pilot-tests were conducted on a random sample of seven included NMAs. Each pilot-test consisted of a facilitated team meeting for feedback and discussion on discrepant items. Upon completion of the pilot-tests, pairs of reviewers (AAV, WZ, JA, SS, PR, CD, JE) independently assessed the first 215 included articles. The remaining 241 included articles were assessed by one reviewer (MP) and verified by a second reviewer (AV, SS). All discrepancies were resolved by a third reviewer (WZ, AAV). ISPOR items that were not applicable to open loop networks (related terms include without a closed loop, star-shaped network, and tree-shaped network) were scored as ‘not applicable'. Items related to heterogeneity were also not applicable to NMAs that used a fixed-effect model and provided a rationale for selecting this model.

### Synthesis

Descriptive analysis using frequencies and percentages were performed to summarize the characteristics of the NMAs. Papers that relied on previous systematic reviews to identify studies for inclusion in the NMA were categorized using content analysis by the lead author (WZ) and verified by the study guarantor (ACT). Journal disciplines were coded by one reviewer (VN) using the Web of Science journal citation reports [28]. The distribution of NMAs by discipline was plotted in a bubble chart using the *ggplot2* library in R software [29, 30]. In order to visualize the frequency of the terms used to describe NMA, a word cloud was created using Wordle [31]. To estimate the time it took to conduct each NMA, we calculated the difference between the initial literature search date and publication date using the *month* and *day* function in Excel 2010. A Pearson correlation coefficient [32] was calculated using Excel 2010 to investigate if a linear relationship existed between duration and quality (according to the AMSTAR score).

## Results

### Literature search

**Fig. 1:**
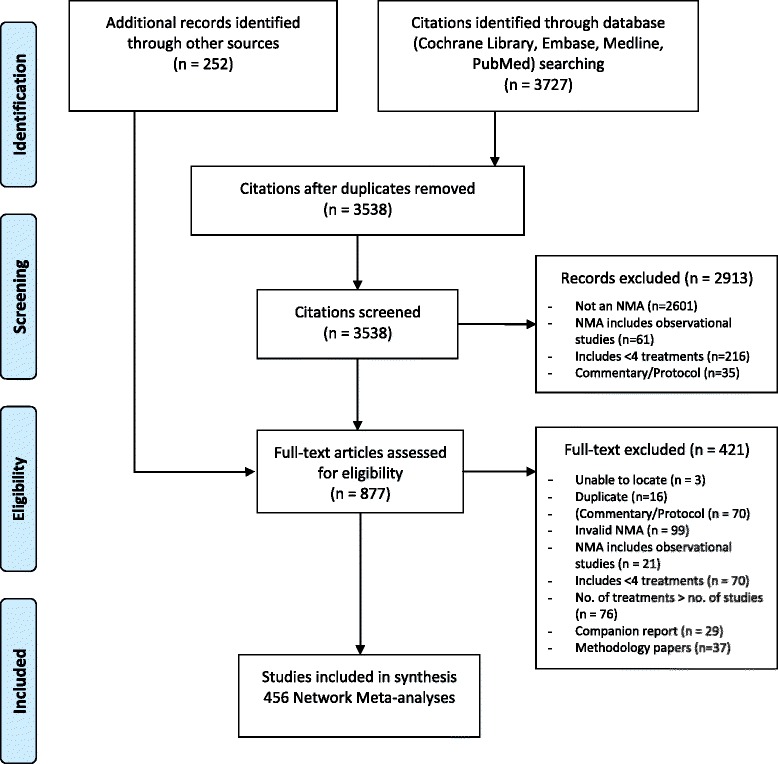
Study flow diagram

The bibliographic database search yielded a total of 3727 citations (Fig. 1). After de-duplication, 3538 unique titles and abstracts were screened and 2913 were excluded. An additional 252 potentially relevant full-texts were identified through supplementary sources. After screening the 877 full-text articles, 456 NMAs fulfilled the eligibility criteria and were included in our scoping review. The full list of included studies can be found in Additional file 1: Appendix 3. Four papers (1%) [33–36] were reports and two papers were non-English publications [37, 38].

### Study characteristics

NMAs in our database were published between July 1999 and February 2015, with 95% (*n* = 432) published after 2006 (Table 1). The majority of the NMAs were conducted in Europe (*n* = 234, 51%), North America (*n* = 140, 31%), and Asia (*n* = 67, 15%). The remaining NMAs were conducted in Central and South America (*n* = 6, 1.3%), Australia and New Zealand (*n* = 7, 1.5%), and Africa (*n* = 2, 0.4%). Eighty percent (*n* = 365) of the NMAs described the knowledge synthesis method as a “systematic review” in either the title or the methods section of the paper, 2% (*n* = 8) described the knowledge synthesis as an “overview of reviews”, less than one percent (*n*=1) used the term “narrative review”, and the remaining 18% (*n* = 82) did not state the type of knowledge synthesis. The median duration from the time of the literature search to publication was 12.5 months (interquartile range (IQR), 7.2–21.8). Twelve percent (*n* = 55) of the NMAs required less than 6 months to be published, 52% (*n* = 238) were published within 6 to 24 months, and 18% (*n* = 81) required more than 24 months to publish. We were not able to estimate duration for the remaining NMAs (18%; *n* = 82) due to a lack of information on the literature search date and/or publication date.

**Table 1:** Study characteristics

Study characteristics (*n* = 456)		Count (%)
Year of publication	1999–2002	3 (0.7)
	2003–2006	21 (4.6)
	2007–2010	77 (16.9)
	2011–2014	306 (67.1)
	2015 (until April)	49 (10.7)
Geographic region	Europe	234 (51.3)
	North America	140 (30.7)
	Asia	67 (14.7)
	Central & South America	6 (1.3)
	Australia & New Zealand	7 (1.5)
	Africa	2 (0.4)
Knowledge synthesis approach	Systematic review	365 (80.0)
	Overview of reviews	8 (1.8)
	Narrative review	1 (0.2)
	Not reported	82 (18.0)
Review duration (month)	<6 months	55 (12.1)
	6–12 months	132 (28.9)
	>12–24 months	106 (23.2)
	>24 months	81 (17.8)
	Not reported	82 (18.0)
Funding	Publicly-sponsored	165 (36.2)
	Industry-sponsored	100 (21.9)
	Non-sponsored	101 (22.1)
	Industry and publicly sponsored	8 (1.8)
	Funding source not reported	82 (18.0)
Full review method reported^a^	Yes	438 (96.1)
	No	18 (3.9)
Number of trials included in review	Median (IQR)	25 (14–48)
Number of trials included in the network	Median (IQR)	21 (13–40)

^a^NMAs without full review method were those with inadequate reporting of review methods (i.e., literature search, study selection, data abstract and quality assessment)

*IQR* interquartile range

Most of the NMAs (*n* = 165, 36%) were publicly sponsored, 22% (*n* = 100) declared industry-sponsorship by a pharmaceutical company or medical device manufacturer, another 22% (*n* = 101) reported that no external funding was received, 2% (*n*=8) of the NMAs reported both industry and public sponsorship, and 18% (*n* = 82) did not disclose any funding information. The median number of RCTs included in the knowledge synthesis was 25 (IQR, 14–48) and the median number of RCTs included in the NMA was 21 (IQR, 13–40).

#### Journal disciplines

**Fig. 2:**
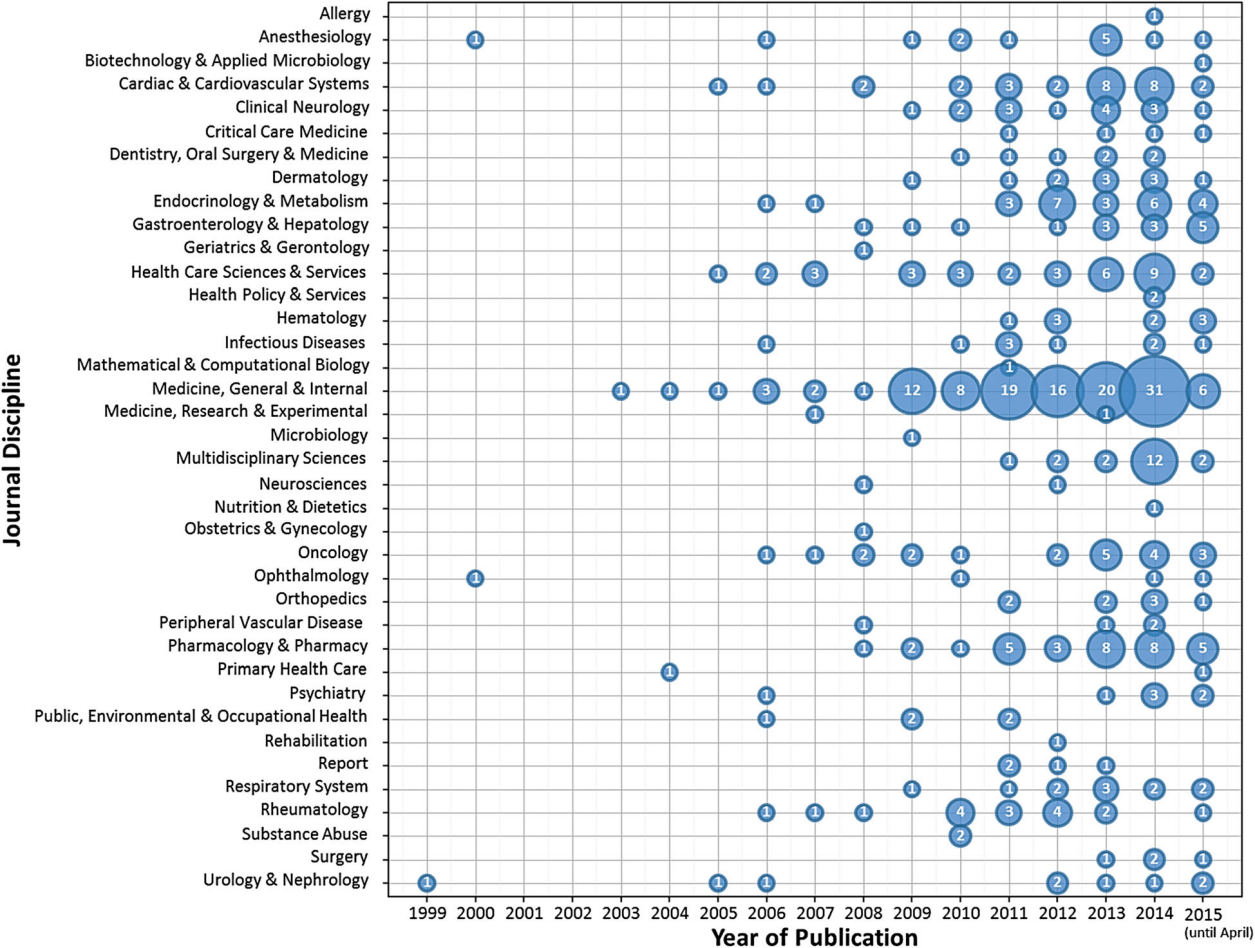
Bubble plot of NMAs published by year and journal discipline (*n* = 456)

The NMAs were published in a broad range of biomedical disciplines (based on the Web of Science journal citation reports). The five most common disciplines with increasing growth overtime were medicine, general and internal (*n* = 121), healthcare sciences and services (*n* = 34), pharmacology and pharmacy (*n* = 33), cardiac and cardiovascular systems (*n* = 29), and endocrinology and metabolism (*n* = 25; Fig. 2).

#### Terminology and cited framework for analysis

The three most commonly used terms to describe a NMA were “network meta-analysis” (*n* = 213, 47%), followed by “mixed-treatment comparisons” (*n* = 108, 24%) and “indirect comparisons” (*n* = 56, 12%; Additional file 1: Appendix 4).

Most NMAs (*n* = 380, 83%) cited at least one previously published methodology paper to guide the analysis, but 76 NMAs (17%) did not cite any methodology paper for the analysis. Of the 123 unique methodology citations, the three most frequently cited papers included a methodology paper on hierarchical Bayesian models for NMAs (*n* = 137, 30%) [10], a paper providing a tutorial on previously described NMA approaches (*n* = 76, 17%) [9], and a paper on a statistical approach to generate indirect evidence as an extension to pairwise meta-analysis (*n* = 71, 16%; Additional file 1: Appendix 5) [7].

### Knowledge synthesis steps

Eighteen papers (4%) did not provide sufficient information on all of the knowledge synthesis steps and therefore could not be characterized. The knowledge synthesis characteristics for the remaining 438 NMAs are summarized in Table 2.

**Table 2:** Knowledge synthesis method characteristics

	Method characteristics (*n* = 438)^a^		Count (%)
A priori protocol and review design	A priori protocol	Use of a protocol mentioned	66 (15.1)
		Published	40 (9.1)
		Registered	25 (5.7)
		Available upon request	6 (1.4)
		Not reported	301 (68.7)
	Research question	Clearly reported	437 (99.8)
		Unclear/inferred	1 (0.2)
	Eligibility criteria	Clearly reported	430 (98.2)
		Unclear/inferred	1 (0.2)
		Not reported	7 (1.6)
Identifying relevant studies	Databases searched	Searched more than one database	407 (92.9)
		Searched only one database	29 (6.6)
		Not reported	2 (0.5)
	Search string	Complete literature search	207 (47.3)
		MeSH terms only	173 (39.5)
		Not reported	58 (13.2)
	Additional search strategy	Scanned references	309 (70.5)
		Grey literature searched	270 (61.6)
		Consulted topic experts	80 (18.3)
		Consulted librarian	67 (15.3)
		Performed updated search	62 (14.2)
		Manually searched selected journals	37 (8.4)
	Limits applied	Limited by study design	291 (66.4)
		Limited by language	147 (33.6)
		Limited by date	135 (30.8)
		Other limits (e.g., age, humans)	129 (29.5)
Study selection	Title & abstract screening	Two or more independent reviewers	285 (65.1)
		One reviewer and one verifier	9 (2.1)
		One reviewer only	16 (3.7)
		Done but unclear number of reviewers	92 (21.0)
		Not reported	36 (8.2)
	Full-text screening	Two or more independent reviewers	282 (64.4)
		One reviewer and one verifier	11 (2.5)
		One reviewer only	7 (1.6)
		Done but unclear number of reviewers	105 (24.0)
		Not reported	33 (7.5)
	Study flow	Completely in PRISMA-like flow diagram	374 (85.4)
		Completely in text/table only	20 (4.6)
		Partially reported	15 (3.4)
		Not reported	29 (6.6)
Data abstraction & quality assessment	Data abstraction	Two or more independent reviewers	238 (54.3)
		One reviewer and one verifier	94 (21.5)
		One reviewer only	8 (1.8)
		Done but unclear number of reviewers	75 (17.1)
		Not reported	23 (5.3)
	Quality appraisal	Two or more independent reviewers	181 (41.3)
		One reviewer and one verifier	21 (4.8)
		One reviewer only	9 (2.1)
		Done but unclear number of reviewers	133 (30.4)
		Not reported	94 (21.5)

^a^18 out of 456 NMAs did not provide details of their knowledge synthesis method

Only 31% (*n* = 137) of the NMAs reported an a priori protocol, but nearly all (*n* = 437, 99.8%) clearly reported their research question and eligibility criteria (*n* = 430, 98%). Ninety-three percent (*n* = 407) of the NMAs searched at least two databases, and 47% (*n* = 207) provided the complete literature search strategy for at least one database. Seventy-one percent (*n* = 309) scanned reference lists of included studies, and 62% (*n* = 270) searched for grey literature (i.e., difficult to locate or unpublished studies [39]). Conference abstracts or proceedings and trial registers were the most common sources of grey literature (133/270, 49%; Additional file 1: Appendix 6). Sixty-six percent (*n* = 291) of the search strategies were limited by study design, 34% (*n* = 147) were limited by language, and 31% (*n* = 135) were limited by date either as a search filter or exclusion criteria.

Duplicate screening by at least two independent reviewers was reported in 65% (*n* = 285) of the NMAs for title and abstract screening, and 64% (*n* = 282) for full-text screening (Table 2). More than half (54%, *n* = 238) of the NMAs completed data abstraction in duplicate, and 41% (*n* = 186) assessed quality in duplicate. The most commonly used tool for risk of bias assessment was the Cochrane Collaboration’s risk-of-bias tool for RCTs (147/345, 42.6%) [40], followed by the Jadad scale [41] (75/345, 22%; Additional file 1: Appendix 7).

### NMAs that relied on previously conducted systematic reviews

Seventy-eight (17%) NMAs relied on previously conducted systematic reviews to identify studies for inclusion in their NMA (Table 3). More than half (*n* = 43) of those NMAs updated the literature search of the systematic review and nearly one-fourth (*n* = 20) used the set of included studies from previous systematic reviews in their analysis (only 2 (10%) of which were from the same group of authors). Eleven NMAs performed an updated literature search with an expanded scope (e.g., included additional drugs), three NMAs used the abstracted data from previous systematic reviews, and one NMA conducted both an updated search of the literature and used the abstracted data from previous reviews.

**Table 3:** Relying on previous reviews (*n* = 456)

NMAs that relied on previous review(s)	Count (%)
Relying on previous reviews (*n* = 456)	
Yes	78 (17.1)
No	378 (82.9)
Themes of use (*n* = 78)	
Updated literature search of previous systematic review(s)	43 (55.1)
Used literature database of previous systematic review(s)	20 (25.6)
Updated and expanded literature search of previous systematic review(s)	11 (14.1)
Used abstracted data of previous systematic review(s)	3 (3.8)
Updated literature search of previous systematic review(s) and used data from previous reviews	1 (1.3)

### AMSTAR assessment

**Fig. 3:**
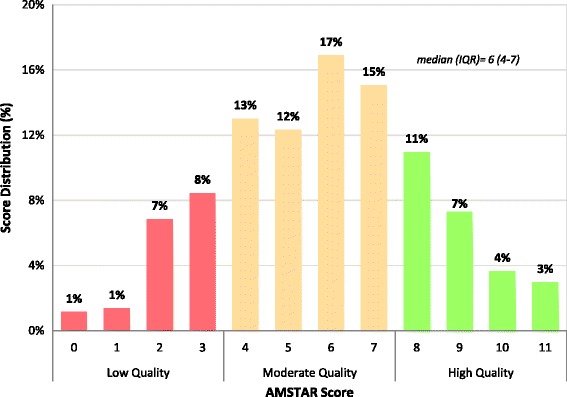
Overall AMSTAR score distribution (*n* = 438)

Our assessments are based on 438 of the NMAs that adequately reported the knowledge synthesis methods. The knowledge synthesis methods used in 25% (*n* = 109) of the NMAs were considered high quality with an AMSTAR score of 8 or above, 57% (*n* = 251) were rated as moderate quality (score 4–7), and the remaining 18% (*n* = 78) were rated as low quality with an AMSTAR score of 3 or less (Fig. 3; Additional file 1: Appendix 8). The overall median AMSTAR score was 6 (IQR, 4–7). The main areas of inadequate reporting that contributed to low AMSTAR scores were lack of a protocol (69%, *n* = 301), lack of a list of excluded studies from full-text screening (82%, *n* = 357), and failing to clearly incorporate quality appraisal results to formulate conclusions either because quality appraisal was not conducted or it was conducted but not incorporated in the interpretation of results (50%, *n* = 221). Publication bias was assessed in only 36% (*n* = 158) of the NMAs and even fewer NMAs (16%, *n* = 68) reported sources of funding of the RCTs included in the knowledge synthesis.

The correlation analysis between duration and overall AMSTAR score found no linear relationship (*r* = 0.014; Additional file 1: Appendix 9). Our graph of temporal trends suggested that the quality of reporting has improved over time with increasing proportions of studies in the “Moderate” and “High” categories (Additional file 1: Appendix 10).

### ISPOR assessment

**Fig. 4:**
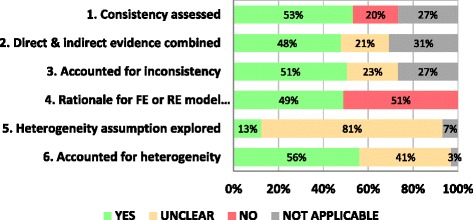
ISPOR assessment by items (*n* = 456)

Fifty-three percent (*n* = 243) of the NMAs either evaluated or discussed consistency in treatment effects, and 48% (*n* = 218) of those networks with consistency conducted a NMA that included both direct and indirect comparisons in the analysis. Fifty-one percent (*n* = 231) accounted for inconsistency or an imbalance in the distribution of treatment effect modifiers across the different types of comparisons in the network of RCTs, if present. Forty-nine percent (*n* = 224) provided a rationale for the choice between a fixed-effect and random-effects model. Only 13% (*n* = 57) discussed the heterogeneity assumption (i.e., choosing between network-specific and comparison-specific heterogeneity) used for the random-effects model, while 81% (*n* = 368) failed to report this item. In the presence of heterogeneity, 56% (*n* = 256) used subgroup, sensitivity or meta-regression analysis to explore heterogeneity, and 41% (*n* = 187) did not mention if heterogeneity was explored (Fig. 4).

One-fourth (*n*=122) of the NMAs were open loop networks, so the network consistency items were scored as ‘not applicable’. Two percent (*n*=9) of the NMAs applied a fixed-effect model and provided a rationale for choosing this model, so heterogeneity items for these were scored as 'not applicable'. However, 4% (*n* = 19) of the NMAs used a fixed-effect model without providing a rationale and more than half of these (*n* = 11) also failed to report any subgroup, sensitivity or meta-regression analysis to explain heterogeneity.

## Discussion

We conducted a comprehensive scoping review of 456 existing NMAs published until February 2015. The earliest year of publication in our database is 1999, and 95% of the NMAs were published after 2006. This suggests that NMA is becoming and established area of knowledge synthesis.

We charted the knowledge synthesis methods used to establish the included studies in the NMAs. Although most authors identified the review type as a systematic review in the title or methods, many shortcuts were observed. For example, one in six NMAs relied on previously conducted systematic reviews to identify RCTs to include in their NMA and a quarter of these did not update the literature search. This may be problematic as numerous relevant and recent studies can be missed, particularly for treatment comparisons that have never been studied previously. Moreover, one-third of the NMAs did not report duplicate screening of citations and full-text articles to identify relevant studies, which is recommended for systematic reviews [42]. Approximately two-thirds of the NMAs searched grey literature, and one-third limited the database search by date and/or language. Failure to search for grey literature increases the likelihood of publication bias, but very few of the included studies formally evaluated the presence of publication bias.

We found that the knowledge synthesis processes underlying the NMAs were of moderate quality, but the quality improved over time. Less than half of the NMAs reported the literature search strategy and 30% reported the use of a protocol. Furthermore, less than a quarter of the NMAs were considered to be of high quality with an AMSTAR score of 8 or greater. Areas for improvement on the AMSTAR tool included use of a protocol, assessment of publication bias, reporting of excluded studies from full-text screening, and reporting the sources of funding of included RCTs. Approximately one-fifth of the NMAs were industry-sponsored, which may pose a potential risk of funding bias [43]. Conversely, areas where the NMAs consistently scored well on the AMSTAR tool included a comprehensive literature search being conducted, characteristics of included studies being reported, and appropriate methods for pairwise meta-analysis being applied.

We used the ISPOR tool to assess the credibility of the analysis of NMAs and found that there is substantial room for improvement. Most authors failed to report the assumptions for heterogeneity used in the random-effects model or explore reasons for heterogeneity when present. Half of the NMAs did not report whether assessment for consistency within closed loops was done, if the NMA combined information from both direct and indirect comparisons or if inconsistencies were accounted for. The recent publication of the PRISMA extension statement for NMAs [15] may lead to improvement in quality of reporting over time. The use of reporting guidelines could increase methodological transparency and uptake of research findings by allowing readers to judge the validity and reliability of studies, and may also reduce waste in biomedical research [44].

There are some limitations to our scoping review that are worth noting. The correlation between duration and AMSTAR score may be biased since we approximated the duration based on the difference between the first literature search date and the date of publication. However, many studies did not clearly report the first literature search date or the publication date, as a result, the duration could not be estimated for approximately one-sixth of the papers. Furthermore, many undocumented lags between completion of the NMA and publication (e.g., journal peer-review process) could inflate this duration. Our analysis was focused primarily on published NMAs (in addition to few identified unpublished reports), thus, our results may not be generalizable to all NMAs, such as those presented at conferences or found in other unpublished formats. However, given the large sample of NMAs in our database, our findings likely represent the overall characteristics of NMAs.

Finally, using the AMSTAR and ISPOR tools to appraise the knowledge synthesis methods and analysis methods for NMAs has some limitations. The AMSTAR tool was designed and validated to assess the methodological quality of systematic reviews of RCTs [26], so it is appropriate for NMAs of RCTs. However, some of the items on the AMSTAR tool can be misinterpreted. For example, item 9 can be misunderstood to suggest that the choice between a fixed-effect and a random-effects model to combine studies be based on a test of homogeneity, which is misguided [45, 46]. The ISPOR tool has been designed to assess networks with at least one closed loop, which is not always applicable to open-loop networks (i.e., adjusted or anchored indirect comparisons). Further, the ISPOR tool assesses whether consistency assessment is discussed, but does not allow for the assessment of approaches that are not valid. It inquires whether consistent networks combine indirect and direct evidence, but does not capture if networks were combined inappropriately. More guidance from the authors of the tool will be beneficial to address these types of scenarios. Finally, some of the NMAs were conducted and published before guidance from AMSTAR or ISPOR existed, so we acknowledge that we are judging those NMAs against standards that were developed much later.

## Conclusion

NMA is becoming an established method and its popularity continues to grow. Our scoping review of 456 NMAs revealed several reporting deficiencies and shortcuts in the knowledge synthesis methods used. This is reflected in the AMSTAR quality rating, with only one-quarter assessed as being high quality. Furthermore, one in six NMAs relied on previously conducted systematic reviews to establish the studies included in the NMA, and a quarter of these did not update the literature search. Improvements in the reporting and conduct of the analytical process for NMAs are also required. Most authors failed to report the assumptions for heterogeneity used in the random-effects model or explore reasons for heterogeneity when present. Since NMAs could be a tremendously useful tool for decision-makers at all levels of the healthcare system (e.g., patients, healthcare providers, policymakers), it is imperative to improve reporting and conduct in order to maximize the transparency, reproducibility, and quality of such studies. Our results suggest that education amongst the research community is required to improve the quality of reporting and methodological quality of published NMAs. Finally, journal editors and peer reviewers should receive adequate training to ensure that only the most methodologically rigorous NMAs are published. Endorsement and implementation of reporting guidelines, such as the PRISMA extension statement for NMAs [16], by the scientific community and journals may improve the completeness of reporting in the future.

## Additional files

Additional file 1: Appendix 1. Study protocol. **Appendix 2.** MEDLINE search strategy. **Appendix 3.** List of included studies (*n* = 456). **Appendix 4.** Network meta-analysis terminology. **Appendix 5.** Methodology papers cited ≥ 10 times. **Appendix 6.** Sources of grey literature searched. **Appendix 7.** Quality appraisal tools used. **Appendix 8.** AMSTAR assessment by items (*n* = 438). **Appendix 9.** Correlation between review duration and AMSTAR quality. **Appendix 10.** AMSTAR quality overtime. (DOCX 651 kb)
Additional file 2: Supplementary data file. (XLSX 449 kb)

## Abbreviations

AMSTAR: A Measurement Tool to Assess Systematic Reviews
ISPOR: International Society of Pharmacoeconomics and Outcomes Research
NMA: network meta-analysis
PRISMA: Preferred Reporting Items for Systematic Reviews and Meta-analyses
RCT: randomised controlled trial
