# Erratum to: Characteristics and knowledge synthesis approach for 456 network meta-analyses: a scoping review

**DOI:** 10.1186/s12916-017-0832-6

**Published:** 2017-03-14

**Authors:** Wasifa Zarin, Areti Angeliki Veroniki, Vera Nincic, Afshin Vafaei, Emily Reynen, Sanober S. Motiwala, Jesmin Antony, Shannon M. Sullivan, Patricia Rios, Caitlin Daly, Joycelyne Ewusie, Maria Petropoulou, Adriani Nikolakopoulou, Anna Chaimani, Georgia Salanti, Sharon E. Straus, Andrea C. Tricco

**Affiliations:** 1 Knowledge Translation Program, Li Ka Shing Knowledge Institute, St. Michael’s Hospital, Toronto, Ontario M5B 1W8 Canada; 2 Department of Hygiene and Epidemiology, University of Ioannina, Ioannina, 45110 Greece; 3 Institute of Social and Preventive Medicine, University of Bern, Bern, Switzerland; 4 Institute of Primary Health Care, University of Bern, Bern, Switzerland; 5 Department of Medicine, Faculty of Medicine, University of Toronto, Toronto, Ontario M5S 1A1 Canada; 6 Epidemiology Division, Dalla Lana School of Public Health, University of Toronto, Toronto, Ontario M5T 3M7 Canada

## Erratum

After the publication of the original article [1], it came to the author’s attention that one of the study’s funding sources was omitted. A Canadian Institutes of Health Research Catalyst Grant (Grant # 342169) should have been acknowledged, so the Funding section of the article should have read as follows:

## Funding

This study was funded by the Canadian Agency for Drugs and Technologies in Health (CADTH), as well as a Canadian Institutes of Health Research Catalyst Grant (Grant # 342169). AAV is funded by the Canadian Institutes of Health Research Banting Postdoctoral Fellowship Program. SES is funded by a Tier 1 Canada Research Chair in Knowledge Translation. ACT is funded by a Tier 2 Canada Research Chair in Knowledge Synthesis.
