# Supplementary material for: Characteristics and knowledge synthesis approach for 456 network meta-analyses: a scoping review
Source: BMC Med. 2017 Jan 5;15:3. doi: 10.1186/s12916-016-0764-6 (PMC5215202; doi:10.1186/s12916-016-0764-6)
Supplement: Additional file 1: Appendix 1. — Study protocol. Appendix 2. MEDLINE search strategy. Appendix 3. List of included studies (n = 456). Appendix 4. Network meta-analysis terminology. Appendix 5. Methodology papers cited ≥ 10 times. Appendix 6. Sources of grey literature searched. Appendix 7. Quality appraisal tools used. Appendix 8. AMSTAR assessment by items (n = 438). Appendix 9. Correlation between review duration and AMSTAR quality. Appendix 10. AMSTAR quality overtime. (DOCX 651 kb) [file 12916_2016_764_MOESM1_ESM.docx]

# Appendix 1. Study protocol

**Type of Review:**

Scoping review

**Search:**

We will use a pre-existing sample of 186 published NMAs that was used to examine statistical methods of NMA in a previous paper.^[[1]](#footnote-1)^

**Study selection:**

Inclusion/Exclusion criteria for the NMA database

1. Include networks that include only RCTs (not observational studies)
2. Include only networks comparing at least 4 treatments (including no treatment, placebo, etc.)
3. Include only networks in which the number of included studies is larger than the number of competing treatments
4. Include only networks that used a valid method for network meta-analysis (e.g. adjusted indirect comparison, hierarchical models, meta-regression models, multivariate meta- analysis model, linear models with 2 stages); for example we excluded networks that used a naive approach like pooling the arms across studies
5. Exclude diagnostic test accuracy studies [i.e., eligible are only networks of treatments/interventions]
6. Exclude animal studies
7. Exclude commentaries
8. Include methodological papers - all examples are eligible and entered as separate NMAs

**Data abstraction:**

We will chart the types of review methods identified and will note the number of NMAs that relied on previously conducted systematic reviews to identify studies for inclusion.

**Data items:**

- Terminology used to describe the review method and the NMA method.
- Is there a protocol available? Where can it be accessed (e.g., Web address, registration information including registration number)?
- Information sources described in the search (e.g., number of databases searched, contact with study authors to identify additional studies, and date last searched).
- Process for selecting studies (e.g., # of independent reviewers who conducted the screening)
- Method of data extraction from reports (e.g., # of independent reviewers who conducted the data abstraction)
- Duration of review (literature search date to publication date used as a proxy if not reported)
- Do the authors list and define the variables for which data were sought (e.g., PICOS, funding sources)?
- Which are the methods used for assessing risk of bias of individual studies and how was this information used in the NMA?
- Method of quality appraisal of included studies (e.g., # of independent reviewers who conducted the appraisal)

**Risk of bias (quality) assessment:**

The quality of included NMAs will be appraised using the AMSTAR (A MeaSurement Tool to Assess Reviews) tool, which provides an overall quality rating on a scale of 0 to 11. A rating of 8 or higher is considered high quality, 4 to 7 is moderate quality and 0 to 3 is low quality. We will also appraise the NMAs using the International Society of Pharmacoeconomics and Outcomes Research (ISPOR) checklist for appraising NMA and a framework for statistical considerations in NMA. All data abstraction and quality appraisal will be conducted by 2 team members, independently.

**Data synthesis:**

We will use quantitative (frequencies) and qualitative (content analysis) methods to summarize the charted data.

**Changes to the Protocol:**

In addition to the existing database, comprehensive literature searches were conducted in MEDLINE, PubMed, EMBASE, and Cochrane Database of Systematic Reviews from inception until April 14, 2015 and references of relevant reviews were scanned. Also, since there were 456 NMAs that met the eligibility criteria, we had to perform data abstraction and quality appraisal for half (n=241) of the studies using one reviewer and one verifier as opposed to two reviewers independently in order to make the workload more manageable.

# Appendix 2. MEDLINE search strategy

**Search Date:** April 14, 2015

**Database:** Ovid MEDLINE(R) In-Process & Other Non-Indexed Citations and Ovid MEDLINE(R) <1946 to Present>

--------------------------------------------------------------------------------

1     (network* adj3 (meta-analy* or metanaly* or metaanaly* or met analy*)).mp.

2     (MTC adj3 (meta-analy* or metanaly* or metaanaly* or met analy*)).mp.

3     (indirect* adj comparison*).mp.

4     (mixed treatment* adj comparison*).mp.

5     (multiple treatment* adj comparison*).mp.

6     (multi-treatment* adj comparison*).mp.

7     (simultaneous* adj comparison*).mp.

8     mixed comparison*.mp. (10)

9     (umbrella adj3 (meta-analy* or metanaly* or metaanaly* or met analy*)).mp.

10     or/1-9

# Appendix 3. List of included studies (n=456)

1. Psaty BM, Smith NL, Siscovick DS, Koepsell TD, Weiss NS, Heckbert SR, et al. Health outcomes associated with antihypertensive therapies used as first-line agents. A systematic review and meta-analysis. JAMA. 1997;277(9):739-45.

2. Djavan B, Marberger M. A meta-analysis on the efficacy and tolerability of alpha1-adrenoceptor antagonists in patients with lower urinary tract symptoms suggestive of benign prostatic obstruction. Eur Urol. 1999;36(1):1-13.

3. Picard P, Tramer MR. Prevention of pain on injection with propofol: a quantitative systematic review. Anesth Analg. 2000;90(4):963-9.

4. Wilhelmus KR. The treatment of herpes simplex virus epithelial keratitis. Trans Am Ophthalmol Soc. 2000;98:505-32.

5. Mason L, Moore RA, Edwards JE, Derry S, McQuay HJ. Topical NSAIDs for acute pain: a meta-analysis. BMC Fam Pract. 2004;5:10.

6. Playford EG, Webster AC, Sorell TC, Craig JC. Antifungal agents for preventing fungal infections in solid organ transplant recipients. Cochrane Database Syst Rev. 2004(3):Cd004291.

7. Wehren LE, Hosking D, Hochberg MC. Putting evidence-based medicine into clinical practice: comparing anti-resorptive agents for the treatment of osteoporosis. Curr Med Res Opin. 2004;20(4):525-31.

8. Biondi-Zoccai GG, Agostoni P, Abbate A, Testa L, Burzotta F, Lotrionte M, et al. Adjusted indirect comparison of intracoronary drug-eluting stents: evidence from a metaanalysis of randomized bare-metal-stent-controlled trials. Int J Cardiol. 2005;100(1):119-23.

9. Brophy JM, Joseph L. Medical decision making with incomplete evidence--choosing a platelet glycoprotein IIbIIIa receptor inhibitor for percutaneous coronary interventions. Med Decis Making. 2005;25(2):222-8.

10. Gafter-Gvili A, Fraser A, Paul M, Leibovici L. Meta-analysis: antibiotic prophylaxis reduces mortality in neutropenic patients. Ann Intern Med. 2005;142(12 Pt 1):979-95.

11. Lapitan MC, Cody DJ, Grant AM. Open retropubic colposuspension for urinary incontinence in women. Cochrane Database Syst Rev. 2005(3):Cd002912.

12. Macfadyen CA, Acuin JM, Gamble C. Topical antibiotics without steroids for chronically discharging ears with underlying eardrum perforations. Cochrane Database Syst Rev. 2005(4):Cd004618.

13. Moore RA, Derry S, McQuay HJ. Indirect comparison of interventions using published randomised trials: systematic review of PDE-5 inhibitors for erectile dysfunction. BMC Urol. 2005;5:18.

14. Richy F, Schacht E, Bruyere O, Ethgen O, Gourlay M, Reginster JY. Vitamin D analogs versus native vitamin D in preventing bone loss and osteoporosis-related fractures: a comparative meta-analysis. Calcif Tissue Int. 2005;76(3):176-86.

15. Berner MM, Kriston L, Harms A. Efficacy of PDE-5-inhibitors for erectile dysfunction. A comparative meta-analysis of fixed-dose regimen randomized controlled trials administering the International Index of Erectile Function in broad-spectrum populations. Int J Impot Res. 2006;18(3):229-35.

16. Brown TJ, Hooper L, Elliott RA, Payne K, Webb R, Roberts C, et al. A comparison of the cost-effectiveness of five strategies for the prevention of non-steroidal anti-inflammatory drug-induced gastrointestinal toxicity: a systematic review with economic modelling. Health Technol Assess. 2006;10(38):iii-iv, xi-xiii, 1-183.

17. Cooper NJ, Sutton AJ, Lu G, Khunti K. Mixed comparison of stroke prevention treatments in individuals with nonrheumatic atrial fibrillation. Arch Intern Med. 2006;166(12):1269-75.

18. Davies L, Brown TJ, Haynes S, Payne K, Elliott RA, McCollum C. Cost-effectiveness of cell salvage and alternative methods of minimising perioperative allogeneic blood transfusion: a systematic review and economic model. Health Technol Assess. 2006;10(44):iii-iv, ix-x, 1-210.

19. Eckert L, Lancon C. Duloxetine compared with fluoxetine and venlafaxine: use of meta-regression analysis for indirect comparisons. BMC Psychiatry. 2006;6:30.

20. Gartlehner G, Hansen RA, Jonas BL, Thieda P, Lohr KN. The comparative efficacy and safety of biologics for the treatment of rheumatoid arthritis: a systematic review and metaanalysis. J Rheumatol. 2006;33(12):2398-408.

21. Jansen JP. Self-monitoring of glucose in type 2 diabetes mellitus: a Bayesian meta-analysis of direct and indirect comparisons. Curr Med Res Opin. 2006;22(4):671-81.

22. Kyrgiou M, Salanti G, Pavlidis N, Paraskevaidis E, Ioannidis JP. Survival benefits with diverse chemotherapy regimens for ovarian cancer: meta-analysis of multiple treatments. J Natl Cancer Inst. 2006;98(22):1655-63.

23. Nelson HD, Vesco KK, Haney E, Fu R, Nedrow A, Miller J, et al. Nonhormonal therapies for menopausal hot flashes: systematic review and meta-analysis. JAMA. 2006;295(17):2057-71.

24. Playford EG, Webster AC, Sorrell TC, Craig JC. Systematic review and meta-analysis of antifungal agents for preventing fungal infections in liver transplant recipients. Eur J Clin Microbiol Infect Dis. 2006;25(9):549-61.

25. Wu P, Wilson K, Dimoulas P, Mills EJ. Effectiveness of smoking cessation therapies: a systematic review and meta-analysis. BMC Public Health. 2006;6:300.

26. Yu CH, Beattie WS. The effects of volatile anesthetics on cardiac ischemic complications and mortality in CABG: a meta-analysis. Can J Anaesth. 2006;53(9):906-18.

27. Zhou Z, Rahme E, Pilote L. Are statins created equal? Evidence from randomized trials of pravastatin, simvastatin, and atorvastatin for cardiovascular disease prevention. Am Heart J. 2006;151(2):273-81.

28. Buscemi N, Vandermeer B, Friesen C, Bialy L, Tubman M, Ospina M, et al. The efficacy and safety of drug treatments for chronic insomnia in adults: a meta-analysis of RCTs. J Gen Intern Med. 2007;22(9):1335-50.

29. Elliott WJ, Meyer PM. Incident diabetes in clinical trials of antihypertensive drugs: a network meta-analysis. Lancet. 2007;369(9557):201-7.

30. Golfinopoulos V, Salanti G, Pavlidis N, Ioannidis JP. Survival and disease-progression benefits with treatment regimens for advanced colorectal cancer: a meta-analysis. Lancet Oncol. 2007;8(10):898-911.

31. Lam SK, Owen A. Combined resynchronisation and implantable defibrillator therapy in left ventricular dysfunction: Bayesian network meta-analysis of randomised controlled trials. BMJ. 2007;335(7626):925.

32. McLeod C, Bagust A, Boland A, Dagenais P, Dickson R, Dundar Y, et al. Adalimumab, etanercept and infliximab for the treatment of ankylosing spondylitis: a systematic review and economic evaluation. Health Technol Assess. 2007;11(28):1-158, iii-iv.

33. Nixon R, Bansback N, Brennan A. The efficacy of inhibiting tumour necrosis factor alpha and interleukin 1 in patients with rheumatoid arthritis: a meta-analysis and adjusted indirect comparisons. Rheumatology (Oxford). 2007;46(7):1140-7.

34. Pollock A, Baer G, Pomeroy V, Langhorne P. Physiotherapy treatment approaches for the recovery of postural control and lower limb function following stroke. Cochrane Database Syst Rev. 2007(1):Cd001920.

35. Soares-Weiser K, Bravo Vergel Y, Beynon S, Dunn G, Barbieri M, Duffy S, et al. A systematic review and economic model of the clinical effectiveness and cost-effectiveness of interventions for preventing relapse in people with bipolar disorder. Health Technol Assess. 2007;11(39):iii-iv, ix-206.

36. Tudur Smith C, Marson AG, Chadwick DW, Williamson PR. Multiple treatment comparisons in epilepsy monotherapy trials. Trials. 2007;8:34.

37. Vestergaard P, Jorgensen NR, Mosekilde L, Schwarz P. Effects of parathyroid hormone alone or in combination with antiresorptive therapy on bone mineral density and fracture risk--a meta-analysis. Osteoporos Int. 2007;18(1):45-57.

38. Abdullah AK, Khan S. Relative oral corticosteroid-sparing effect of 7 inhaled corticosteroids in chronic asthma: a meta-analysis. Ann Allergy Asthma Immunol. 2008;101(1):74-81.

39. Chou R, Carson S, Chan BK. Pegylated interferons for chronic hepatitis C virus infection: an indirect analysis of randomized trials. J Viral Hepat. 2008;15(8):551-70.

40. Coleman CI, Baker WL, Kluger J, White CM. Antihypertensive medication and their impact on cancer incidence: a mixed treatment comparison meta-analysis of randomized controlled trials. J Hypertens. 2008;26(4):622-9.

41. Eisenberg MJ, Filion KB, Yavin D, Belisle P, Mottillo S, Joseph L, et al. Pharmacotherapies for smoking cessation: a meta-analysis of randomized controlled trials. CMAJ. 2008;179(2):135-44.

42. Hansen RA, Gartlehner G, Webb AP, Morgan LC, Moore CG, Jonas DE. Efficacy and safety of donepezil, galantamine, and rivastigmine for the treatment of Alzheimer's disease: a systematic review and meta-analysis. Clin Interv Aging. 2008;3(2):211-25.

43. Hansen RA, Gaynes BN, Gartlehner G, Moore CG, Tiwari R, Lohr KN. Efficacy and tolerability of second-generation antidepressants in social anxiety disorder. Int Clin Psychopharmacol. 2008;23(3):170-9.

44. Hofmeyr GJ, Gulmezoglu AM. Misoprostol for the prevention and treatment of postpartum haemorrhage. Best Pract Res Clin Obstet Gynaecol. 2008;22(6):1025-41.

45. Mauri D, Polyzos NP, Salanti G, Pavlidis N, Ioannidis JP. Multiple-treatments meta-analysis of chemotherapy and targeted therapies in advanced breast cancer. J Natl Cancer Inst. 2008;100(24):1780-91.

46. Mills EJ, Rachlis B, Wu P, Devereaux PJ, Arora P, Perri D. Primary prevention of cardiovascular mortality and events with statin treatments: a network meta-analysis involving more than 65,000 patients. J Am Coll Cardiol. 2008;52(22):1769-81.

47. Peterson K, McDonagh MS, Fu R. Comparative benefits and harms of competing medications for adults with attention-deficit hyperactivity disorder: a systematic review and indirect comparison meta-analysis. Psychopharmacology (Berl). 2008;197(1):1-11.

48. Sultana A, Ghaneh P, Cunningham D, Starling N, Neoptolemos JP, Smith CT. Gemcitabine based combination chemotherapy in advanced pancreatic cancer-indirect comparison. BMC Cancer. 2008;8:192.

49. Thijs V, Lemmens R, Fieuws S. Network meta-analysis: simultaneous meta-analysis of common antiplatelet regimens after transient ischaemic attack or stroke. Eur Heart J. 2008;29(9):1086-92.

50. Ara R, Pandor A, Stevens J, Rees A, Rafia R. Early high-dose lipid-lowering therapy to avoid cardiac events: a systematic review and economic evaluation. Health Technol Assess. 2009;13(34):1-74, 5-118.

51. Baker WL, Baker EL, Coleman CI. Pharmacologic treatments for chronic obstructive pulmonary disease: a mixed-treatment comparison meta-analysis. Pharmacotherapy. 2009;29(8):891-905.

52. Bansback N, Sizto S, Sun H, Feldman S, Willian MK, Anis A. Efficacy of systemic treatments for moderate to severe plaque psoriasis: systematic review and meta-analysis. Dermatology. 2009;219(3):209-18.

53. Cipriani A, Furukawa TA, Salanti G, Geddes JR, Higgins JP, Churchill R, et al. Comparative efficacy and acceptability of 12 new-generation antidepressants: a multiple-treatments meta-analysis. Lancet. 2009;373(9665):746-58.

54. Edwards SJ, Clarke MJ, Wordsworth S, Welton NJ. Carbapenems versus other beta-lactams in the treatment of hospitalised patients with infection: a mixed treatment comparison. Curr Med Res Opin. 2009;25(1):251-61.

55. Edwards SJ, Lind T, Lundell L, Das R. Systematic review: standard- and double-dose proton pump inhibitors for the healing of severe erosive oesophagitis -- a mixed treatment comparison of randomized controlled trials. Aliment Pharmacol Ther. 2009;30(6):547-56.

56. Edwards SJ, Smith CJ. Tolerability of atypical antipsychotics in the treatment of adults with schizophrenia or bipolar disorder: a mixed treatment comparison of randomized controlled trials. Clin Ther. 2009;31 Pt 1:1345-59.

57. Golfinopoulos V, Pentheroudakis G, Salanti G, Nearchou AD, Ioannidis JP, Pavlidis N. Comparative survival with diverse chemotherapy regimens for cancer of unknown primary site: multiple-treatments meta-analysis. Cancer Treat Rev. 2009;35(7):570-3.

58. Goudswaard AN, Furlong NJ, Rutten GE, Stolk RP, Valk GD. Insulin monotherapy versus combinations of insulin with oral hypoglycaemic agents in patients with type 2 diabetes mellitus. Cochrane Database Syst Rev. 2009(4):Cd003418.

59. Hawkins N, Scott DA, Woods BS, Thatcher N. No study left behind: a network meta-analysis in non-small-cell lung cancer demonstrating the importance of considering all relevant data. Value Health. 2009;12(6):996-1003.

60. Hofmeyr GJ, Gulmezoglu AM, Novikova N, Linder V, Ferreira S, Piaggio G. Misoprostol to prevent and treat postpartum haemorrhage: a systematic review and meta-analysis of maternal deaths and dose-related effects. Bull World Health Organ. 2009;87(9):666-77.

61. Jansen JP, Bergman GJ, Huels J, Olson M. Prevention of vertebral fractures in osteoporosis: mixed treatment comparison of bisphosphonate therapies. Curr Med Res Opin. 2009;25(8):1861-8.

62. Kotze A, Scally A, Howell S. Efficacy and safety of different techniques of paravertebral block for analgesia after thoracotomy: a systematic review and metaregression. Br J Anaesth. 2009;103(5):626-36.

63. Mills EJ, Perri D, Cooper C, Nachega JB, Wu P, Tleyjeh I, et al. Antifungal treatment for invasive Candida infections: a mixed treatment comparison meta-analysis. Ann Clin Microbiol Antimicrob. 2009;8:23.

64. Mills EJ, Rachlis B, O'Regan C, Thabane L, Perri D. Metastatic renal cell cancer treatments: an indirect comparison meta-analysis. BMC Cancer. 2009;9:34.

65. Mills EJ, Wu P, Spurden D, Ebbert JO, Wilson K. Efficacy of pharmacotherapies for short-term smoking abstinance: a systematic review and meta-analysis. Harm Reduct J. 2009;6:25.

66. Puhan MA, Bachmann LM, Kleijnen J, Ter Riet G, Kessels AG. Inhaled drugs to reduce exacerbations in patients with chronic obstructive pulmonary disease: a network meta-analysis. BMC Med. 2009;7:2.

67. Quilici S, Chancellor J, Lothgren M, Simon D, Said G, Le TK, et al. Meta-analysis of duloxetine vs. pregabalin and gabapentin in the treatment of diabetic peripheral neuropathic pain. BMC Neurol. 2009;9:6.

68. Singh JA, Christensen R, Wells GA, Suarez-Almazor ME, Buchbinder R, Lopez-Olivo MA, et al. A network meta-analysis of randomized controlled trials of biologics for rheumatoid arthritis: a Cochrane overview. CMAJ. 2009;181(11):787-96.

69. Singh JA, Christensen R, Wells GA, Suarez-Almazor ME, Buchbinder R, Lopez-Olivo MA, et al. Biologics for rheumatoid arthritis: an overview of Cochrane reviews. Cochrane Database Syst Rev. 2009(4):Cd007848.

70. Strassmann R, Bausch B, Spaar A, Kleijnen J, Braendli O, Puhan MA. Smoking cessation interventions in COPD: a network meta-analysis of randomised trials. Eur Respir J. 2009;34(3):634-40.

71. Trikalinos TA, Alsheikh-Ali AA, Tatsioni A, Nallamothu BK, Kent DM. Percutaneous coronary interventions for non-acute coronary artery disease: a quantitative 20-year synopsis and a network meta-analysis. Lancet. 2009;373(9667):911-8.

72. van der Valk R, Webers CA, Lumley T, Hendrikse F, Prins MH, Schouten JS. A network meta-analysis combined direct and indirect comparisons between glaucoma drugs to rank effectiveness in lowering intraocular pressure. J Clin Epidemiol. 2009;62(12):1279-83.

73. Welton NJ, Caldwell DM, Adamopoulos E, Vedhara K. Mixed treatment comparison meta-analysis of complex interventions: psychological interventions in coronary heart disease. Am J Epidemiol. 2009;169(9):1158-65.

74. Bergman GJ, Hochberg MC, Boers M, Wintfeld N, Kielhorn A, Jansen JP. Indirect comparison of tocilizumab and other biologic agents in patients with rheumatoid arthritis and inadequate response to disease-modifying antirheumatic drugs. Semin Arthritis Rheum. 2010;39(6):425-41.

75. Dakin H, Fidler C, Harper C. Mixed treatment comparison meta-analysis evaluating the relative efficacy of nucleos(t)ides for treatment of nucleos(t)ide-naive patients with chronic hepatitis B. Value Health. 2010;13(8):934-45.

76. Delahoy P, Thompson S, Marschner IC. Pregabalin versus gabapentin in partial epilepsy: a meta-analysis of dose-response relationships. BMC Neurol. 2010;10:104.

77. Hauser W, Petzke F, Sommer C. Comparative efficacy and harms of duloxetine, milnacipran, and pregabalin in fibromyalgia syndrome. J Pain. 2010;11(6):505-21.

78. Imamura M, Abrams P, Bain C, Buckley B, Cardozo L, Cody J, et al. Systematic review and economic modelling of the effectiveness and cost-effectiveness of non-surgical treatments for women with stress urinary incontinence. Health Technol Assess. 2010;14(40):1-188, iii-iv.

79. McDaid C, Maund E, Rice S, Wright K, Jenkins B, Woolacott N. Paracetamol and selective and non-selective non-steroidal anti-inflammatory drugs (NSAIDs) for the reduction of morphine-related side effects after major surgery: a systematic review. Health Technol Assess. 2010;14(17):1-153, iii-iv.

80. Meader N. A comparison of methadone, buprenorphine and alpha(2) adrenergic agonists for opioid detoxification: a mixed treatment comparison meta-analysis. Drug Alcohol Depend. 2010;108(1-2):110-4.

81. Middleton LJ, Champaneria R, Daniels JP, Bhattacharya S, Cooper KG, Hilken NH, et al. Hysterectomy, endometrial destruction, and levonorgestrel releasing intrauterine system (Mirena) for heavy menstrual bleeding: systematic review and meta-analysis of data from individual patients. BMJ. 2010;341:c3929.

82. Orme M, Collins S, Dakin H, Kelly S, Loftus J. Mixed treatment comparison and meta-regression of the efficacy and safety of prostaglandin analogues and comparators for primary open-angle glaucoma and ocular hypertension. Curr Med Res Opin. 2010;26(3):511-28.

83. Owen A. Antithrombotic treatment for the primary prevention of stroke in patients with non valvular atrial fibrillation: a reappraisal of the evidence and network meta analysis. Int J Cardiol. 2010;142(3):218-23.

84. Phung OJ, Scholle JM, Talwar M, Coleman CI. Effect of noninsulin antidiabetic drugs added to metformin therapy on glycemic control, weight gain, and hypoglycemia in type 2 diabetes. JAMA. 2010;303(14):1410-8.

85. Piscione F, Piccolo R, Cassese S, Galasso G, De Rosa R, D'Andrea C, et al. Effect of drug-eluting stents in patients with acute ST-segment elevation myocardial infarction undergoing percutaneous coronary intervention: a meta-analysis of randomised trials and an adjusted indirect comparison. EuroIntervention. 2010;5(7):853-60.

86. Roskell NS, Lip GY, Noack H, Clemens A, Plumb JM. Treatments for stroke prevention in atrial fibrillation: a network meta-analysis and indirect comparisons versus dabigatran etexilate. Thromb Haemost. 2010;104(6):1106-15.

87. Stowe R, Ives N, Clarke CE, Deane K, Wheatley K, Gray R, et al. Evaluation of the efficacy and safety of adjuvant treatment to levodopa therapy in Parkinson s disease patients with motor complications. Cochrane Database Syst Rev. 2010(7):Cd007166.

88. Tu YK, Woolston A, Faggion CM, Jr. Do bone grafts or barrier membranes provide additional treatment effects for infrabony lesions treated with enamel matrix derivatives? A network meta-analysis of randomized-controlled trials. J Clin Periodontol. 2010;37(1):59-79.

89. Uthman OA, Abdulmalik J. Comparative efficacy and acceptability of pharmacotherapeutic agents for anxiety disorders in children and adolescents: a mixed treatment comparison meta-analysis. Curr Med Res Opin. 2010;26(1):53-9.

90. Vissers D, Stam W, Nolte T, Lenre M, Jansen J. Efficacy of intranasal fentanyl spray versus other opioids for breakthrough pain in cancer. Curr Med Res Opin. 2010;26(5):1037-45.

91. Wandel S, Juni P, Tendal B, Nuesch E, Villiger PM, Welton NJ, et al. Effects of glucosamine, chondroitin, or placebo in patients with osteoarthritis of hip or knee: network meta-analysis. BMJ. 2010;341:c4675.

92. Wang H, Huang T, Jing J, Jin J, Wang P, Yang M, et al. Effectiveness of different central venous catheters for catheter-related infections: a network meta-analysis. J Hosp Infect. 2010;76(1):1-11.

93. Woo G, Tomlinson G, Nishikawa Y, Kowgier M, Sherman M, Wong DK, et al. Tenofovir and entecavir are the most effective antiviral agents for chronic hepatitis B: a systematic review and Bayesian meta-analyses. Gastroenterology. 2010;139(4):1218-29.

94. Anothaisintawee T, Attia J, Nickel JC, Thammakraisorn S, Numthavaj P, McEvoy M, et al. Management of chronic prostatitis/chronic pelvic pain syndrome: a systematic review and network meta-analysis. JAMA. 2011;305(1):78-86.

95. Baldwin D, Woods R, Lawson R, Taylor D. Efficacy of drug treatments for generalised anxiety disorder: systematic review and meta-analysis. BMJ. 2011;342:d1199.

96. Bangalore S, Kumar S, Kjeldsen SE, Makani H, Grossman E, Wetterslev J, et al. Antihypertensive drugs and risk of cancer: network meta-analyses and trial sequential analyses of 324,168 participants from randomised trials. Lancet Oncol. 2011;12(1):65-82.

97. Bekkering GE, Soares-Weiser K, Reid K, Kessels AG, Dahan A, Treede RD, et al. Can morphine still be considered to be the standard for treating chronic pain? A systematic review including pair-wise and network meta-analyses. Curr Med Res Opin. 2011;27(7):1477-91.

98. Blanchard P, Hill C, Guihenneuc-Jouyaux C, Baey C, Bourhis J, Pignon JP. Mixed treatment comparison meta-analysis of altered fractionated radiotherapy and chemotherapy in head and neck cancer. J Clin Epidemiol. 2011;64(9):985-92.

99. Bottomley JM, Taylor RS, Ryttov J. The effectiveness of two-compound formulation calcipotriol and betamethasone dipropionate gel in the treatment of moderately severe scalp psoriasis: a systematic review of direct and indirect evidence. Curr Med Res Opin. 2011;27(1):251-68.

100. Brodszky V. Effectiveness of biological treatments based on ACR70 response in rheumatoid arthritis: indirect comparison and meta-regression using Bayes-model. Orv Hetil. 2011;152(23):919-28.

101. Choy E, Marshall D, Gabriel ZL, Mitchell SA, Gylee E, Dakin HA. A systematic review and mixed treatment comparison of the efficacy of pharmacological treatments for fibromyalgia. Semin Arthritis Rheum. 2011;41(3):335-45.e6.

102. Cipriani A, Barbui C, Salanti G, Rendell J, Brown R, Stockton S, et al. Comparative efficacy and acceptability of antimanic drugs in acute mania: a multiple-treatments meta-analysis. Lancet. 2011;378(9799):1306-15.

103. Costa J, Fareleira F, Ascencao R, Borges M, Sampaio C, Vaz-Carneiro A. Clinical comparability of the new antiepileptic drugs in refractory partial epilepsy: a systematic review and meta-analysis. Epilepsia. 2011;52(7):1280-91.

104. Dakin HA, Welton NJ, Ades AE, Collins S, Orme M, Kelly S. Mixed treatment comparison of repeated measurements of a continuous endpoint: an example using topical treatments for primary open-angle glaucoma and ocular hypertension. Stat Med. 2011;30(20):2511-35.

105. Danchin N, Marzilli M, Parkhomenko A, Ribeiro JP. Efficacy comparison of trimetazidine with therapeutic alternatives in stable angina pectoris: a network meta-analysis. Cardiology. 2011;120(2):59-72.

106. Devine EB, Alfonso-Cristancho R, Sullivan SD. Effectiveness of biologic therapies for rheumatoid arthritis: an indirect comparisons approach. Pharmacotherapy. 2011;31(1):39-51.

107. Freemantle N, Lafuente-Lafuente C, Mitchell S, Eckert L, Reynolds M. Mixed treatment comparison of dronedarone, amiodarone, sotalol, flecainide, and propafenone, for the management of atrial fibrillation. Europace. 2011;13(3):329-45.

108. Freemantle N, Tharmanathan P, Herbrecht R. Systematic review and mixed treatment comparison of randomized evidence for empirical, pre-emptive and directed treatment strategies for invasive mould disease. J Antimicrob Chemother. 2011;66 Suppl 1:i25-35.

109. Gartlehner G, Hansen RA, Morgan LC, Thaler K, Lux L, Van Noord M, et al. Comparative benefits and harms of second-generation antidepressants for treating major depressive disorder: an updated meta-analysis. Ann Intern Med. 2011;155(11):772-85.

110. Gross JL, Kramer CK, Leitao CB, Hawkins N, Viana LV, Schaan BD, et al. Effect of antihyperglycemic agents added to metformin and a sulfonylurea on glycemic control and weight gain in type 2 diabetes: a network meta-analysis. Ann Intern Med. 2011;154(10):672-9.

111. Gurusamy KS, Pissanou T, Pikhart H, Vaughan J, Burroughs AK, Davidson BR. Methods to decrease blood loss and transfusion requirements for liver transplantation. Cochrane Database Syst Rev. 2011(12):Cd009052.

112. Guyot P, Taylor P, Christensen R, Pericleous L, Poncet C, Lebmeier M, et al. Abatacept with methotrexate versus other biologic agents in treatment of patients with active rheumatoid arthritis despite methotrexate: a network meta-analysis. Arthritis Res Ther. 2011;13(6):R204.

113. Hartling L, Fernandes RM, Bialy L, Milne A, Johnson D, Plint A, et al. Steroids and bronchodilators for acute bronchiolitis in the first two years of life: systematic review and meta-analysis. BMJ. 2011;342:d1714.

114. Hauser W, Petzke F, Uceyler N, Sommer C. Comparative efficacy and acceptability of amitriptyline, duloxetine and milnacipran in fibromyalgia syndrome: a systematic review with meta-analysis. Rheumatology (Oxford). 2011;50(3):532-43.

115. Hopkins RB, Goeree R, Pullenayegum E, Adachi JD, Papaioannou A, Xie F, et al. The relative efficacy of nine osteoporosis medications for reducing the rate of fractures in post-menopausal women. BMC Musculoskelet Disord. 2011;12:209.

116. Ibrahim T, Qureshi A, Sutton AJ, Dias JJ. Surgical versus nonsurgical treatment of acute minimally displaced and undisplaced scaphoid waist fractures: pairwise and network meta-analyses of randomized controlled trials. J Hand Surg Am. 2011;36(11):1759-68.e1.

117. Jalota L, Kalira V, George E, Shi YY, Hornuss C, Radke O, et al. Prevention of pain on injection of propofol: systematic review and meta-analysis. BMJ. 2011;342:d1110.

118. Jansen JP, Bergman GJ, Huels J, Olson M. The efficacy of bisphosphonates in the prevention of vertebral, hip, and nonvertebral-nonhip fractures in osteoporosis: a network meta-analysis. Semin Arthritis Rheum. 2011;40(4):275-84.e1-2.

119. Kessler TM, Bachmann LM, Minder C, Lohrer D, Umbehr M, Schunemann HJ, et al. Adverse event assessment of antimuscarinics for treating overactive bladder: a network meta-analytic approach. PLoS One. 2011;6(2):e16718.

120. Klemp M, Tvete IF, Skomedal T, Gaasemyr J, Natvig B, Aursnes I. A review and Bayesian meta-analysis of clinical efficacy and adverse effects of 4 atypical neuroleptic drugs compared with haloperidol and placebo. J Clin Psychopharmacol. 2011;31(6):698-704.

121. Launois R, Avouac B, Berenbaum F, Blin O, Bru I, Fautrel B, et al. Comparison of certolizumab pegol with other anticytokine agents for treatment of rheumatoid arthritis: a multiple-treatment Bayesian metaanalysis. J Rheumatol. 2011;38(5):835-45.

122. Makani H, Bangalore S, Romero J, Wever-Pinzon O, Messerli FH. Effect of renin-angiotensin system blockade on calcium channel blocker-associated peripheral edema. Am J Med. 2011;124(2):128-35.

123. Maratea D, Fadda V, Trippoli S, Messori A. Prevention of venous thromboembolism after major orthopedic surgery: indirect comparison of three new oral anticoagulants. J Thromb Haemost. 2011;9(9):1868-70.

124. Maund E, McDaid C, Rice S, Wright K, Jenkins B, Woolacott N. Paracetamol and selective and non-selective non-steroidal anti-inflammatory drugs for the reduction in morphine-related side-effects after major surgery: a systematic review. Br J Anaesth. 2011;106(3):292-7.

125. McIntosh B, Cameron C, Singh SR, Yu C, Ahuja T, Welton NJ, et al. Second-line therapy in patients with type 2 diabetes inadequately controlled with metformin monotherapy: a systematic review and mixed-treatment comparison meta-analysis. Open Med. 2011;5(1):e35-48.

126. Mills EJ, Druyts E, Ghement I, Puhan MA. Pharmacotherapies for chronic obstructive pulmonary disease: a multiple treatment comparison meta-analysis. Clin Epidemiol. 2011;3:107-29.

127. Mills EJ, Wu P, Chong G, Ghement I, Singh S, Akl EA, et al. Efficacy and safety of statin treatment for cardiovascular disease: a network meta-analysis of 170,255 patients from 76 randomized trials. QJM. 2011;104(2):109-24.

128. Padwal R, Klarenbach S, Wiebe N, Birch D, Karmali S, Manns B, et al. Bariatric surgery: a systematic review and network meta-analysis of randomized trials. Obes Rev. 2011;12(8):602-21.

129. Phung OJ, Kahn SR, Cook DJ, Murad MH. Dosing frequency of unfractionated heparin thromboprophylaxis: a meta-analysis. Chest. 2011;140(2):374-81.

130. Phung OJ, Sood NA, Sill BE, Coleman CI. Oral anti-diabetic drugs for the prevention of Type 2 diabetes. Diabet Med. 2011;28(8):948-64.

131. Rheims S, Perucca E, Cucherat M, Ryvlin P. Factors determining response to antiepileptic drugs in randomized controlled trials. A systematic review and meta-analysis. Epilepsia. 2011;52(2):219-33.

132. Riemsma R, Forbes C, Harker J, Worthy G, Misso K, Schafer M, et al. Systematic review of tapentadol in chronic severe pain. Curr Med Res Opin. 2011;27(10):1907-30.

133. Roskell NS, Beard SM, Zhao Y, Le TK. A meta-analysis of pain response in the treatment of fibromyalgia. Pain Pract. 2011;11(6):516-27.

134. Salliot C, Finckh A, Katchamart W, Lu Y, Sun Y, Bombardier C, et al. Indirect comparisons of the efficacy of biological antirheumatic agents in rheumatoid arthritis in patients with an inadequate response to conventional disease-modifying antirheumatic drugs or to an anti-tumour necrosis factor agent: a meta-analysis. Ann Rheum Dis. 2011;70(2):266-71.

135. Sciarretta S, Palano F, Tocci G, Baldini R, Volpe M. Antihypertensive treatment and development of heart failure in hypertension: a Bayesian network meta-analysis of studies in patients with hypertension and high cardiovascular risk. Arch Intern Med. 2011;171(5):384-94.

136. Selph S, Carson S, Fu R, Thakurta S, Low A, McDonagh M. Drug Class Reviews. Drug Class Review: Neuropathic Pain: Final Update 1 Report. Portland (OR): Oregon Health & Science University; 2011.

137. Singh JA, Wells GA, Christensen R, Tanjong Ghogomu E, Maxwell L, Macdonald JK, et al. Adverse effects of biologics: a network meta-analysis and Cochrane overview. Cochrane Database Syst Rev. 2011(2):Cd008794.

138. Smith B, Peterson K, Fu R, McDonagh M, Thakurta S. Drug Class Reviews. Drug Class Review: Drugs for Fibromyalgia: Final Original Report. Portland (OR): Oregon Health & Science University; 2011.

139. Squires H, Simpson E, Meng Y, Harnan S, Stevens J, Wong R, et al. A systematic review and economic evaluation of cilostazol, naftidrofuryl oxalate, pentoxifylline and inositol nicotinate for the treatment of intermittent claudication in people with peripheral arterial disease. Health Technol Assess. 2011;15(40):1-210.

140. Stowe R, Ives N, Clarke CE, Handley K, Furmston A, Deane K, et al. Meta-analysis of the comparative efficacy and safety of adjuvant treatment to levodopa in later Parkinson's disease. Mov Disord. 2011;26(4):587-98.

141. Trelle S, Reichenbach S, Wandel S, Hildebrand P, Tschannen B, Villiger PM, et al. Cardiovascular safety of non-steroidal anti-inflammatory drugs: network meta-analysis. BMJ. 2011;342:c7086.

142. Tropeano AI, Saleh N, Hawajri N, Macquin-Mavier I, Maison P. Do all antihypertensive drugs improve carotid intima-media thickness? A network meta-analysis of randomized controlled trials. Fundam Clin Pharmacol. 2011;25(3):395-404.

143. Turkstra E, Ng SK, Scuffham PA. A mixed treatment comparison of the short-term efficacy of biologic disease modifying anti-rheumatic drugs in established rheumatoid arthritis. Curr Med Res Opin. 2011;27(10):1885-97.

144. Van den Bruel A, Gailly J, Devriese S, Welton NJ, Shortt AJ, Vrijens F. The protective effect of ophthalmic viscoelastic devices on endothelial cell loss during cataract surgery: a meta-analysis using mixed treatment comparisons. Br J Ophthalmol. 2011;95(1):5-10.

145. Vieira MC, Kumar RN, Jansen JP. Comparative effectiveness of efavirenz, protease inhibitors, and raltegravir-based regimens as first-line treatment for HIV-infected adults: a mixed treatment comparison. HIV Clin Trials. 2011;12(4):175-89.

146. Virgili G, Novielli N, Menchini F, Murro V, Giacomelli G. Pharmacological treatments for neovascular age-related macular degeneration: can mixed treatment comparison meta-analysis be useful? Curr Drug Targets. 2011;12(2):212-20.

147. Wiebe N, Padwal R, Field C, Marks S, Jacobs R, Tonelli M. A systematic review on the effect of sweeteners on glycemic response and clinically relevant outcomes. BMC Med. 2011;9:123.

148. Wong MC, Clarkson J, Glenny AM, Lo EC, Marinho VC, Tsang BW, et al. Cochrane reviews on the benefits/risks of fluoride toothpastes. J Dent Res. 2011;90(5):573-9.

149. Ziogas DC, Voulgarelis M, Zintzaras E. A network meta-analysis of randomized controlled trials of induction treatments in acute myeloid leukemia in the elderly. Clin Ther. 2011;33(3):254-79.

150. Alberton M, Wu P, Druyts E, Briel M, Mills EJ. Adverse events associated with individual statin treatments for cardiovascular disease: an indirect comparison meta-analysis. QJM. 2012;105(2):145-57.

151. Ara R, Blake L, Gray L, Hernandez M, Crowther M, Dunkley A, et al. What is the clinical effectiveness and cost-effectiveness of using drugs in treating obese patients in primary care? A systematic review. Health Technol Assess. 2012;16(5):iii-xiv, 1-195.

152. Asseburg C, Peura P, Oksanen T, Turunen J, Purmonen T, Martikainen J. Cost-effectiveness of oral triptans for acute migraine: mixed treatment comparison. Int J Technol Assess Health Care. 2012;28(4):382-9.

153. Baker EL, Coleman CI, Reinhart KM, Phung OJ, Kugelman L, Chen W, et al. Effect of Biologic Agents on Non-PASI Outcomes in Moderate-to-Severe Plaque Psoriasis: Systematic Review and Meta-Analyses. Dermatol Ther (Heidelb). 2012;2(1):9.

154. Bally M, Dendukuri N, Sinclair A, Ahern SP, Poisson M, Brophy J. A network meta-analysis of antibiotics for treatment of hospitalised patients with suspected or proven meticillin-resistant Staphylococcus aureus infection. Int J Antimicrob Agents. 2012;40(6):479-95.

155. Bangalore S, Kumar S, Fusaro M, Amoroso N, Attubato MJ, Feit F, et al. Short- and long-term outcomes with drug-eluting and bare-metal coronary stents: a mixed-treatment comparison analysis of 117 762 patient-years of follow-up from randomized trials. Circulation. 2012;125(23):2873-91.

156. Bangalore S, Kumar S, Fusaro M, Amoroso N, Kirtane AJ, Byrne RA, et al. Outcomes with various drug eluting or bare metal stents in patients with diabetes mellitus: mixed treatment comparison analysis of 22,844 patient years of follow-up from randomised trials. BMJ. 2012;345:e5170.

157. Bash LD, Buono JL, Davies GM, Martin A, Fahrbach K, Phatak H, et al. Systematic review and meta-analysis of the efficacy of cardioversion by vernakalant and comparators in patients with atrial fibrillation. Cardiovasc Drugs Ther. 2012;26(2):167-79.

158. Buser N, Ivic S, Kessler TM, Kessels AG, Bachmann LM. Efficacy and adverse events of antimuscarinics for treating overactive bladder: network meta-analyses. Eur Urol. 2012;62(6):1040-60.

159. Chang KV, Chen SY, Chen WS, Tu YK, Chien KL. Comparative effectiveness of focused shock wave therapy of different intensity levels and radial shock wave therapy for treating plantar fasciitis: a systematic review and network meta-analysis. Arch Phys Med Rehabil. 2012;93(7):1259-68.

160. Chen YF, Madan J, Welton N, Yahaya I, Aveyard P, Bauld L, et al. Effectiveness and cost-effectiveness of computer and other electronic aids for smoking cessation: a systematic review and network meta-analysis. Health Technol Assess. 2012;16(38):1-205, iii-v.

161. Cohen A, Drost P, Marchant N, Mitchell S, Orme M, Rublee D, et al. The efficacy and safety of pharmacological prophylaxis of venous thromboembolism following elective knee or hip replacement: systematic review and network meta-analysis. Clin Appl Thromb Hemost. 2012;18(6):611-27.

162. Cooper NJ, Kendrick D, Achana F, Dhiman P, He Z, Wynn P, et al. Network meta-analysis to evaluate the effectiveness of interventions to increase the uptake of smoke alarms. Epidemiol Rev. 2012;34:32-45.

163. Cope S, Kraemer M, Zhang J, Capkun-Niggli G, Jansen JP. Efficacy of indacaterol 75 mug versus fixed-dose combinations of formoterol-budesonide or salmeterol-fluticasone for COPD: a network meta-analysis. Int J Chron Obstruct Pulmon Dis. 2012;7:415-20.

164. Cope S, Zhang J, Williams J, Jansen JP. Efficacy of once-daily indacaterol 75 mug relative to alternative bronchodilators in COPD: a study level and a patient level network meta-analysis. BMC Pulm Med. 2012;12:29.

165. Daniels JP, Middleton LJ, Champaneria R, Khan KS, Cooper K, Mol BW, et al. Second generation endometrial ablation techniques for heavy menstrual bleeding: network meta-analysis. BMJ. 2012;344:e2564.

166. Del Santo F, Maratea D, Fadda V, Trippoli S, Messori A. Treatments for relapsing-remitting multiple sclerosis: summarising current information by network meta-analysis. Eur J Clin Pharmacol. 2012;68(4):441-8.

167. Dequen P, Lorigan P, Jansen JP, van Baardewijk M, Ouwens MJ, Kotapati S. Systematic review and network meta-analysis of overall survival comparing 3 mg/kg ipilimumab with alternative therapies in the management of pretreated patients with unresectable stage III or IV melanoma. Oncologist. 2012;17(11):1376-85.

168. Desai RJ, Hansen RA, Rao JK, Wilkins TM, Harden EA, Yuen A, et al. Mixed treatment comparison of the treatment discontinuations of biologic disease-modifying antirheumatic drugs in adults with rheumatoid arthritis. Ann Pharmacother. 2012;46(11):1491-505.

169. Donahue KE, Jonas DE, Hansen RA, Roubey R, Jonas B, Lux LJ, et al. AHRQ Comparative Effectiveness Reviews. Drug Therapy for Rheumatoid Arthritis in Adults: An Update. Rockville (MD): Agency for Healthcare Research and Quality (US); 2012.

170. Dumville JC, Soares MO, O'Meara S, Cullum N. Systematic review and mixed treatment comparison: dressings to heal diabetic foot ulcers. Diabetologia. 2012;55(7):1902-10.

171. Dunkley AJ, Charles K, Gray LJ, Camosso-Stefinovic J, Davies MJ, Khunti K. Effectiveness of interventions for reducing diabetes and cardiovascular disease risk in people with metabolic syndrome: systematic review and mixed treatment comparison meta-analysis. Diabetes Obes Metab. 2012;14(7):616-25.

172. Fox BD, Kahn SR, Langleben D, Eisenberg MJ, Shimony A. Efficacy and safety of novel oral anticoagulants for treatment of acute venous thromboembolism: direct and adjusted indirect meta-analysis of randomised controlled trials. BMJ. 2012;345:e7498.

173. Fretheim A, Odgaard-Jensen J, Brors O, Madsen S, Njolstad I, Norheim OF, et al. Comparative effectiveness of antihypertensive medication for primary prevention of cardiovascular disease: systematic review and multiple treatments meta-analysis. BMC Med. 2012;10:33.

174. Gallego-Galisteo M, Villa-Rubio A, Alegre-del Rey E, Marquez-Fernandez E, Ramos-Baez JJ. Indirect comparison of biological treatments in refractory rheumatoid arthritis. J Clin Pharm Ther. 2012;37(3):301-7.

175. Gomez-Outes A, Terleira-Fernandez AI, Suarez-Gea ML, Vargas-Castrillon E. Dabigatran, rivaroxaban, or apixaban versus enoxaparin for thromboprophylaxis after total hip or knee replacement: systematic review, meta-analysis, and indirect treatment comparisons. BMJ. 2012;344:e3675.

176. Gray LJ, Cooper N, Dunkley A, Warren FC, Ara R, Abrams K, et al. A systematic review and mixed treatment comparison of pharmacological interventions for the treatment of obesity. Obes Rev. 2012;13(6):483-98.

177. Guyot P, Taylor PC, Christensen R, Pericleous L, Drost P, Eijgelshoven I, et al. Indirect treatment comparison of abatacept with methotrexate versus other biologic agents for active rheumatoid arthritis despite methotrexate therapy in the United kingdom. J Rheumatol. 2012;39(6):1198-206.

178. Haas DM, Caldwell DM, Kirkpatrick P, McIntosh JJ, Welton NJ. Tocolytic therapy for preterm delivery: systematic review and network meta-analysis. BMJ. 2012;345:e6226.

179. Harenberg J, Marx S, Dahl OE, Marder VJ, Schulze A, Wehling M, et al. Interpretation of endpoints in a network meta-analysis of new oral anticoagulants following total hip or total knee replacement surgery. Thromb Haemost. 2012;108(5):903-12.

180. Hutton B, Joseph L, Fergusson D, Mazer CD, Shapiro S, Tinmouth A. Risks of harms using antifibrinolytics in cardiac surgery: systematic review and network meta-analysis of randomised and observational studies. BMJ. 2012;345:e5798.

181. Lang SH, Manning N, Armstrong N, Misso K, Allen A, Di Nisio M, et al. Treatment with tirofiban for acute coronary syndrome (ACS): a systematic review and network analysis. Curr Med Res Opin. 2012;28(3):351-70.

182. Lin VW, Ringold S, Devine EB. Comparison of Ustekinumab With Other Biological Agents for the Treatment of Moderate to Severe Plaque Psoriasis: A Bayesian Network Meta-analysis. Arch Dermatol. 2012;148(12):1403-10.

183. Littlewood KJ, Higashi K, Jansen JP, Capkun-Niggli G, Balp MM, Doering G, et al. A network meta-analysis of the efficacy of inhaled antibiotics for chronic Pseudomonas infections in cystic fibrosis. J Cyst Fibros. 2012;11(5):419-26.

184. Liu SC, Tu YK, Chien MN, Chien KL. Effect of antidiabetic agents added to metformin on glycaemic control, hypoglycaemia and weight change in patients with type 2 diabetes: a network meta-analysis. Diabetes Obes Metab. 2012;14(9):810-20.

185. Mak KH. Coronary and mortality risk of novel oral antithrombotic agents: a meta-analysis of large randomised trials. BMJ Open. 2012;2(5).

186. Martyn-St James M, Glanville J, McCool R, Duffy S, Cooper J, Hugel P, et al. The efficacy and safety of retigabine and other adjunctive treatments for refractory partial epilepsy: a systematic review and indirect comparison. Seizure. 2012;21(9):665-78.

187. Mhaskar R, Redzepovic J, Wheatley K, Clark OA, Miladinovic B, Glasmacher A, et al. Bisphosphonates in multiple myeloma: a network meta-analysis. Cochrane Database Syst Rev. 2012;5:Cd003188.

188. Mills EJ, Wu P, Lockhart I, Thorlund K, Puhan M, Ebbert JO. Comparisons of high-dose and combination nicotine replacement therapy, varenicline, and bupropion for smoking cessation: a systematic review and multiple treatment meta-analysis. Ann Med. 2012;44(6):588-97.

189. Murad MH, Drake MT, Mullan RJ, Mauck KF, Stuart LM, Lane MA, et al. Clinical review. Comparative effectiveness of drug treatments to prevent fragility fractures: a systematic review and network meta-analysis. J Clin Endocrinol Metab. 2012;97(6):1871-80.

190. Ni RH, Tang HL, Zhai SD, Li ZL. Multiple treatments for infantile rotavirus enteritis: a network meta-analysis. World J Gastroenterol. 2012;20(5):438-43.

191. Palmerini T, Biondi-Zoccai G, Della Riva D, Stettler C, Sangiorgi D, D'Ascenzo F, et al. Stent thrombosis with drug-eluting and bare-metal stents: evidence from a comprehensive network meta-analysis. Lancet. 2012;379(9824):1393-402.

192. Pichenot M, Deuffic-Burban S, Cuzin L, Yazdanpanah Y. Efficacy of new antiretroviral drugs in treatment-experienced HIV-infected patients: a systematic review and meta-analysis of recent randomized controlled trials. HIV Med. 2012;13(3):148-55.

193. Ramsberg J, Asseburg C, Henriksson M. Effectiveness and cost-effectiveness of antidepressants in primary care: a multiple treatment comparison meta-analysis and cost-effectiveness model. PLoS One. 2012;7(8):e42003.

194. Reich K, Burden AD, Eaton JN, Hawkins NS. Efficacy of biologics in the treatment of moderate to severe psoriasis: a network meta-analysis of randomized controlled trials. Br J Dermatol. 2012;166(1):179-88.

195. Roskell NS, Zimovetz EA, Rycroft CE, Eckert BJ, Tyas DA. Annualized relapse rate of first-line treatments for multiple sclerosis: a meta-analysis, including indirect comparisons versus fingolimod. Curr Med Res Opin. 2012;28(5):767-80.

196. Schmitz S, Adams R, Walsh CD, Barry M, FitzGerald O. A mixed treatment comparison of the efficacy of anti-TNF agents in rheumatoid arthritis for methotrexate non-responders demonstrates differences between treatments: a Bayesian approach. Ann Rheum Dis. 2012;71(2):225-30.

197. Stam W, Jansen J, Taylor S. Efficacy of etoricoxib, celecoxib, lumiracoxib, non-selective NSAIDs, and acetaminophen in osteoarthritis: a mixed treatment comparison. Open Rheumatol J. 2012;6:6-20.

198. Steiner S, Moertl D, Chen L, Coyle D, Wells GA. Network meta-analysis of prasugrel, ticagrelor, high- and standard-dose clopidogrel in patients scheduled for percutaneous coronary interventions. Thromb Haemost. 2012;108(2):318-27.

199. Sun F, Yu K, Wu S, Zhang Y, Yang Z, Shi L, et al. Cardiovascular safety and glycemic control of glucagon-like peptide-1 receptor agonists for type 2 diabetes mellitus: a pairwise and network meta-analysis. Diabetes Res Clin Pract. 2012;98(3):386-95.

200. Sun F, Yu K, Yang Z, Wu S, Zhang Y, Shi L, et al. Impact of GLP-1 receptor agonists on major gastrointestinal disorders for type 2 diabetes mellitus: a mixed treatment comparison meta-analysis. Exp Diabetes Res. 2012;2012:230624.

201. Tang DH, Malone DC. A network meta-analysis on the efficacy of serotonin type 3 receptor antagonists used in adults during the first 24 hours for postoperative nausea and vomiting prophylaxis. Clin Ther. 2012;34(2):282-94.

202. Thakkinstian A, Attia J, Anothaisintawee T, Nickel JC. alpha-blockers, antibiotics and anti-inflammatories have a role in the management of chronic prostatitis/chronic pelvic pain syndrome. BJU Int. 2012;110(7):1014-22.

203. Thorlund K, Druyts E, Avina-Zubieta JA, Mills EJ. Anti-tumor necrosis factor (TNF) drugs for the treatment of psoriatic arthritis: an indirect comparison meta-analysis. Biologics. 2012;6:417-27.

204. Trinquart L, Abbe A, Ravaud P. Impact of reporting bias in network meta-analysis of antidepressant placebo-controlled trials. PLoS One. 2012;7(4):e35219.

205. Tu YK, Needleman I, Chambrone L, Lu HK, Faggion CM, Jr. A Bayesian network meta-analysis on comparisons of enamel matrix derivatives, guided tissue regeneration and their combination therapies. J Clin Periodontol. 2012;39(3):303-14.

206. Vejakama P, Thakkinstian A, Lertrattananon D, Ingsathit A, Ngarmukos C, Attia J. Reno-protective effects of renin-angiotensin system blockade in type 2 diabetic patients: a systematic review and network meta-analysis. Diabetologia. 2012;55(3):566-78.

207. Wolff RF, Aune D, Truyers C, Hernandez AV, Misso K, Riemsma R, et al. Systematic review of efficacy and safety of buprenorphine versus fentanyl or morphine in patients with chronic moderate to severe pain. Curr Med Res Opin. 2012;28(5):833-45.

208. Zagmutt FJ, Tarrants ML. Indirect comparisons of adverse events and dropout rates in early Parkinson's disease trials of pramipexole, ropinirole, and rasagiline. Int J Neurosci. 2012;122(7):345-53.

209. Akshintala VS, Hutfless SM, Colantuoni E, Kim KJ, Khashab MA, Li T, et al. Systematic review with network meta-analysis: pharmacological prophylaxis against post-ERCP pancreatitis. Aliment Pharmacol Ther. 2013;38(11-12):1325-37.

210. Assiri A, Al-Majzoub O, Kanaan AO, Donovan JL, Silva M. Mixed treatment comparison meta-analysis of aspirin, warfarin, and new anticoagulants for stroke prevention in patients with nonvalvular atrial fibrillation. Clin Ther. 2013;35(7):967-84.e2.

211. Bangalore S, Amoroso N, Fusaro M, Kumar S, Feit F. Outcomes with various drug-eluting or bare metal stents in patients with ST-segment-elevation myocardial infarction: a mixed treatment comparison analysis of trial level data from 34 068 patient-years of follow-up from randomized trials. Circ Cardiovasc Interv. 2013;6(4):378-90.

212. Bangalore S, Toklu B, Amoroso N, Fusaro M, Kumar S, Hannan EL, et al. Bare metal stents, durable polymer drug eluting stents, and biodegradable polymer drug eluting stents for coronary artery disease: mixed treatment comparison meta-analysis. BMJ. 2013;347:f6625.

213. Barth J, Munder T, Gerger H, Nuesch E, Trelle S, Znoj H, et al. Comparative efficacy of seven psychotherapeutic interventions for patients with depression: a network meta-analysis. PLoS Med. 2013;10(5):e1001454.

214. Biondi-Zoccai G, Malavasi V, D'Ascenzo F, Abbate A, Agostoni P, Lotrionte M, et al. Comparative effectiveness of novel oral anticoagulants for atrial fibrillation: evidence from pair-wise and warfarin-controlled network meta-analyses. HSR Proc Intensive Care Cardiovasc Anesth. 2013;5(1):40-54.

215. Bodalia PN, Grosso AM, Sofat R, Macallister RJ, Smeeth L, Dhillon S, et al. Comparative efficacy and tolerability of anti-epileptic drugs for refractory focal epilepsy: systematic review and network meta-analysis reveals the need for long term comparator trials. Br J Clin Pharmacol. 2013;76(5):649-67.

216. Braun SR, Gregor B, Tran US. Comparing bona fide psychotherapies of depression in adults with two meta-analytical approaches. PLoS One. 2013;8(6):e68135.

217. Brown T, Pilkington G, Bagust A, Boland A, Oyee J, Tudur-Smith C, et al. Clinical effectiveness and cost-effectiveness of first-line chemotherapy for adult patients with locally advanced or metastatic non-small cell lung cancer: a systematic review and economic evaluation. Health Technol Assess. 2013;17(31):1-278.

218. Buti J, Baccini M, Nieri M, La Marca M, Pini-Prato GP. Bayesian network meta-analysis of root coverage procedures: ranking efficacy and identification of best treatment. J Clin Periodontol. 2013;40(4):372-86.

219. Cahill K, Stevens S, Perera R, Lancaster T. Pharmacological interventions for smoking cessation: an overview and network meta-analysis. Cochrane Database Syst Rev. 2013;5:Cd009329.

220. Carroll C, Hummel S, Leaviss J, Ren S, Stevens JW, Everson-Hock E, et al. Clinical effectiveness and cost-effectiveness of minimally invasive techniques to manage varicose veins: a systematic review and economic evaluation. Health Technol Assess. 2013;17(48):i-xvi, 1-141.

221. Castellucci LA, Cameron C, Le Gal G, Rodger MA, Coyle D, Wells PS, et al. Efficacy and safety outcomes of oral anticoagulants and antiplatelet drugs in the secondary prevention of venous thromboembolism: systematic review and network meta-analysis. BMJ. 2013;347:f5133.

222. Cawston H, Davie A, Paget MA, Skljarevski V, Happich M. Efficacy of duloxetine versus alternative oral therapies: an indirect comparison of randomised clinical trials in chronic low back pain. Eur Spine J. 2013;22(9):1996-2009.

223. Chatterjee S, Biondi-Zoccai G, Abbate A, D'Ascenzo F, Castagno D, Van Tassell B, et al. Benefits of beta blockers in patients with heart failure and reduced ejection fraction: network meta-analysis. BMJ. 2013;346:f55.

224. Chatterjee S, Sardar P, Biondi-Zoccai G, Kumbhani DJ. New oral anticoagulants and the risk of intracranial hemorrhage: traditional and Bayesian meta-analysis and mixed treatment comparison of randomized trials of new oral anticoagulants in atrial fibrillation. JAMA Neurol. 2013;70(12):1486-90.

225. Cooper C, Lester R, Thorlund K, Druyts E, El Khoury AC, Yaya S, et al. Direct-acting antiviral therapies for hepatitis C genotype 1 infection: a multiple treatment comparison meta-analysis. QJM. 2013;106(2):153-63.

226. Cope S, Donohue JF, Jansen JP, Kraemer M, Capkun-Niggli G, Baldwin M, et al. Comparative efficacy of long-acting bronchodilators for COPD: a network meta-analysis. Respir Res. 2013;14:100.

227. Cope S, Ouwens MJ, Jansen JP, Schmid P. Progression-free survival with fulvestrant 500 mg and alternative endocrine therapies as second-line treatment for advanced breast cancer: a network meta-analysis with parametric survival models. Value Health. 2013;16(2):403-17.

228. Corbett MS, Rice SJ, Madurasinghe V, Slack R, Fayter DA, Harden M, et al. Acupuncture and other physical treatments for the relief of pain due to osteoarthritis of the knee: network meta-analysis. Osteoarthritis Cartilage. 2013;21(9):1290-8.

229. Datto C, Hellmund R, Siddiqui MK. Efficacy and tolerability of naproxen/esomeprazole magnesium tablets compared with non-specific NSAIDs and COX-2 inhibitors: a systematic review and network analyses. Open Access Rheumatology: Research & Reviews. 2013;5(26).

230. Delea TE, Amdahl J, Chit A, Amonkar MM. Cost-effectiveness of lapatinib plus letrozole in her2-positive, hormone receptor-positive metastatic breast cancer in Canada. Curr Oncol. 2013;20(5):e371-87.

231. Dong YH, Lin HH, Shau WY, Wu YC, Chang CH, Lai MS. Comparative safety of inhaled medications in patients with chronic obstructive pulmonary disease: systematic review and mixed treatment comparison meta-analysis of randomised controlled trials. Thorax. 2013;68(1):48-56.

232. Dumville JC, McFarlane E, Edwards P, Lipp A, Holmes A. Preoperative skin antiseptics for preventing surgical wound infections after clean surgery. Cochrane Database Syst Rev. 2013;3:Cd003949.

233. Fang Y, Ding Y, Guo Q, Xing J, Long Y, Zong Z. Radioiodine therapy for patients with differentiated thyroid cancer after thyroidectomy: direct comparison and network meta-analyses. J Endocrinol Invest. 2013;36(10):896-902.

234. Filippini G, Del Giovane C, Vacchi L, D'Amico R, Di Pietrantonj C, Beecher D, et al. Immunomodulators and immunosuppressants for multiple sclerosis: a network meta-analysis. Cochrane Database Syst Rev. 2013;6:Cd008933.

235. Fountoulakis KN, Veroniki AA, Siamouli M, Moller HJ. No role for initial severity on the efficacy of antidepressants: results of a multi-meta-analysis. Ann Gen Psychiatry. 2013;12(1):26.

236. Freemantle N, Cooper C, Diez-Perez A, Gitlin M, Radcliffe H, Shepherd S, et al. Results of indirect and mixed treatment comparison of fracture efficacy for osteoporosis treatments: a meta-analysis. Osteoporos Int. 2013;24(1):209-17.

237. Gagne JJ, Bykov K, Choudhry NK, Toomey TJ, Connolly JG, Avorn J. Effect of smoking on comparative efficacy of antiplatelet agents: systematic review, meta-analysis, and indirect comparison. BMJ. 2013;347:f5307.

238. Galvan-Banqueri M, Marin Gil R, Santos Ramos B, Bautista Paloma FJ. Biological treatments for moderate-to-severe psoriasis: indirect comparison. J Clin Pharm Ther. 2013;38(2):121-30.

239. Gao L, Xia L, Zhao FL, Li SC. Clinical efficacy and safety of the newer antiepileptic drugs as adjunctive treatment in adults with refractory partial-onset epilepsy: a meta-analysis of randomized placebo-controlled trials. Epilepsy Res. 2013;103(1):31-44.

240. Goralczyk AD, Cameron S, Amanzada A. Treatment of chronic HCV genotype 1 infection with telaprevir: a Bayesian mixed treatment comparison of fixed-length and response-guided treatment regimens in treatment-naive and -experienced patients. BMC Gastroenterol. 2013;13:148.

241. Gupta AK, Paquet M. Network meta-analysis of the outcome 'participant complete clearance' in nonimmunosuppressed participants of eight interventions for actinic keratosis: a follow-up on a Cochrane review. Br J Dermatol. 2013;169(2):250-9.

242. Hadjigeorgiou GM, Doxani C, Miligkos M, Ziakas P, Bakalos G, Papadimitriou D, et al. A network meta-analysis of randomized controlled trials for comparing the effectiveness and safety profile of treatments with marketing authorization for relapsing multiple sclerosis. J Clin Pharm Ther. 2013;38(6):433-9.

243. Hoaglin DC, Filonenko A, Glickman ME, Wasiak R, Gidwani R. Use of mixed-treatment-comparison methods in estimating efficacy of treatments for heavy menstrual bleeding. Eur J Med Res. 2013;18:17.

244. Howell N, Senanayake E, Freemantle N, Pagano D. Putting the record straight on aprotinin as safe and effective: results from a mixed treatment meta-analysis of trials of aprotinin. J Thorac Cardiovasc Surg. 2013;145(1):234-40.

245. Jonas DE, Cusack K, Forneris CA, Wilkins TM, Sonis J, Middleton JC, et al. AHRQ Comparative Effectiveness Reviews. Psychological and Pharmacological Treatments for Adults With Posttraumatic Stress Disorder (PTSD). Rockville (MD): Agency for Healthcare Research and Quality (US); 2013.

246. Karabis A, Lindner L, Mocarski M, Huisman E, Greening A. Comparative efficacy of aclidinium versus glycopyrronium and tiotropium, as maintenance treatment of moderate to severe COPD patients: a systematic review and network meta-analysis. Int J Chron Obstruct Pulmon Dis. 2013;8:405-23.

247. Khan N, Shah D, Tongbram V, Verdian L, Hawkins N. The efficacy and tolerability of perampanel and other recently approved anti-epileptic drugs for the treatment of refractory partial onset seizure: a systematic review and Bayesian network meta-analysis. Curr Med Res Opin. 2013;29(8):1001-13.

248. Krogh TP, Bartels EM, Ellingsen T, Stengaard-Pedersen K, Buchbinder R, Fredberg U, et al. Comparative effectiveness of injection therapies in lateral epicondylitis: a systematic review and network meta-analysis of randomized controlled trials. Am J Sports Med. 2013;41(6):1435-46.

249. Kwak HJ, Kim JY, Kim YB, Min SK, Moon BK, Kim JY. Pharmacological prevention of rocuronium-induced injection pain or withdrawal movements: a meta-analysis. J Anesth. 2013;27(5):742-9.

250. Kwok CS, Pradhan S, Yeong JK, Loke YK. Relative effects of two different enoxaparin regimens as comparators against newer oral anticoagulants: meta-analysis and adjusted indirect comparison. Chest. 2013;144(2):593-600.

251. Landoni G, Greco T, Biondi-Zoccai G, Nigro Neto C, Febres D, Pintaudi M, et al. Anaesthetic drugs and survival: a Bayesian network meta-analysis of randomized trials in cardiac surgery. Br J Anaesth. 2013;111(6):886-96.

252. Leucht S, Cipriani A, Spineli L, Mavridis D, Orey D, Richter F, et al. Comparative efficacy and tolerability of 15 antipsychotic drugs in schizophrenia: a multiple-treatments meta-analysis. Lancet. 2013;382(9896):951-62.

253. Levi Marpillat N, Macquin-Mavier I, Tropeano AI, Bachoud-Levi AC, Maison P. Antihypertensive classes, cognitive decline and incidence of dementia: a network meta-analysis. J Hypertens. 2013;31(6):1073-82.

254. Liao WC, Chien KL, Lin YL, Wu MS, Lin JT, Wang HP, et al. Adjuvant treatments for resected pancreatic adenocarcinoma: a systematic review and network meta-analysis. Lancet Oncol. 2013;14(11):1095-103.

255. Lin PY, Cheng YW, Chu CY, Chien KL, Lin CP, Tu YK. In-office treatment for dentin hypersensitivity: a systematic review and network meta-analysis. J Clin Periodontol. 2013;40(1):53-64.

256. Liu J, Dong J, Wang L, Su Y, Yan P, Sun S. Comparative efficacy and acceptability of antidepressants in Parkinson's disease: a network meta-analysis. PLoS One. 2013;8(10):e76651.

257. Malloy RJ, Kanaan AO, Silva MA, Donovan JL. Evaluation of antiplatelet agents for secondary prevention of stroke using mixed treatment comparison meta-analysis. Clin Ther. 2013;35(10):1490-500.e7.

258. Mavranezouli I, Meader N, Cape J, Kendall T. The cost effectiveness of pharmacological treatments for generalized anxiety disorder. Pharmacoeconomics. 2013;31(4):317-33.

259. Mealing S, Barcena L, Hawkins N, Clark J, Eaton V, Hirji I, et al. The relative efficacy of imatinib, dasatinib and nilotinib for newly diagnosed chronic myeloid leukemia: a systematic review and network meta-analysis. Exp Hematol Oncol. 2013;2(1):5.

260. Meissner K, Fassler M, Rucker G, Kleijnen J, Hrobjartsson A, Schneider A, et al. Differential effectiveness of placebo treatments: a systematic review of migraine prophylaxis. JAMA Intern Med. 2013;173(21):1941-51.

261. Migliore A, Broccoli S, Massafra U, Cassol M, Frediani B. Ranking antireabsorptive agents to prevent vertebral fractures in postmenopausal osteoporosis by mixed treatment comparison meta-analysis. Eur Rev Med Pharmacol Sci. 2013;17(5):658-67.

262. Naci H, Brugts J, Ades T. Comparative tolerability and harms of individual statins: a study-level network meta-analysis of 246 955 participants from 135 randomized, controlled trials. Circ Cardiovasc Qual Outcomes. 2013;6(4):390-9.

263. Naci H, Brugts JJ, Fleurence R, Ades AE. Dose-comparative effects of different statins on serum lipid levels: a network meta-analysis of 256,827 individuals in 181 randomized controlled trials. Eur J Prev Cardiol. 2013;20(4):658-70.

264. Naci H, Brugts JJ, Fleurence R, Ades AE. Comparative effects of statins on major cerebrovascular events: a multiple-treatments meta-analysis of placebo-controlled and active-comparator trials. QJM. 2013;106(4):299-306.

265. Naci H, Brugts JJ, Fleurence R, Tsoi B, Toor H, Ades AE. Comparative benefits of statins in the primary and secondary prevention of major coronary events and all-cause mortality: a network meta-analysis of placebo-controlled and active-comparator trials. Eur J Prev Cardiol. 2013;20(4):641-57.

266. Naci H, Ioannidis JP. Comparative effectiveness of exercise and drug interventions on mortality outcomes: metaepidemiological study. BMJ. 2013;347:f5577.

267. Naudet F, Millet B, Charlier P, Reymann JM, Maria AS, Falissard B. Which placebo to cure depression? A thought-provoking network meta-analysis. BMC Med. 2013;11:230.

268. Navarese EP, Buffon A, Andreotti F, Kozinski M, Welton N, Fabiszak T, et al. Meta-analysis of impact of different types and doses of statins on new-onset diabetes mellitus. Am J Cardiol. 2013;111(8):1123-30.

269. Navarese EP, Tandjung K, Claessen B, Andreotti F, Kowalewski M, Kandzari DE, et al. Safety and efficacy outcomes of first and second generation durable polymer drug eluting stents and biodegradable polymer biolimus eluting stents in clinical practice: comprehensive network meta-analysis. BMJ. 2013;347:f6530.

270. Nuesch E, Hauser W, Bernardy K, Barth J, Juni P. Comparative efficacy of pharmacological and non-pharmacological interventions in fibromyalgia syndrome: network meta-analysis. Ann Rheum Dis. 2013;72(6):955-62.

271. Palmerini T, Biondi-Zoccai G, Della Riva D, Mariani A, Sabate M, Valgimigli M, et al. Clinical outcomes with drug-eluting and bare-metal stents in patients with ST-segment elevation myocardial infarction: evidence from a comprehensive network meta-analysis. J Am Coll Cardiol. 2013;62(6):496-504.

272. Palmieri C, Fullarton JR, Brown J. Comparative efficacy of bisphosphonates in metastatic breast and prostate cancer and multiple myeloma: a mixed-treatment meta-analysis. Clin Cancer Res. 2013;19(24):6863-72.

273. Pandor A, Gomersall T, Stevens JW, Wang J, Al-Mohammad A, Bakhai A, et al. Remote monitoring after recent hospital discharge in patients with heart failure: a systematic review and network meta-analysis. Heart. 2013;99(23):1717-26.

274. Rashiq S, Vandermeer B, Abou-Setta AM, Beaupre LA, Jones CA, Dryden DM. Efficacy of supplemental peripheral nerve blockade for hip fracture surgery: multiple treatment comparison. Can J Anaesth. 2013;60(3):230-43.

275. Ribeiro RA, Ziegelmann PK, Duncan BB, Stella SF, da Costa Vieira JL, Restelatto LM, et al. Impact of statin dose on major cardiovascular events: a mixed treatment comparison meta-analysis involving more than 175,000 patients. Int J Cardiol. 2013;166(2):431-9.

276. Rotta I, Ziegelmann PK, Otuki MF, Riveros BS, Bernardo NL, Correr CJ. Efficacy of topical antifungals in the treatment of dermatophytosis: a mixed-treatment comparison meta-analysis involving 14 treatments. JAMA Dermatol. 2013;149(3):341-9.

277. Rudroju N, Bansal D, Talakokkula ST, Gudala K, Hota D, Bhansali A, et al. Comparative efficacy and safety of six antidepressants and anticonvulsants in painful diabetic neuropathy: a network meta-analysis. Pain Physician. 2013;16(6):E705-14.

278. Samarasekera EJ, Sawyer L, Wonderling D, Tucker R, Smith CH. Topical therapies for the treatment of plaque psoriasis: systematic review and network meta-analyses. Br J Dermatol. 2013;168(5):954-67.

279. Schoenberg MB, Marx S, Kersten JF, Rosch T, Belle S, Kahler G, et al. Laparoscopic Heller myotomy versus endoscopic balloon dilatation for the treatment of achalasia: a network meta-analysis. Ann Surg. 2013;258(6):943-52.

280. Scott DA, Boye KS, Timlin L, Clark JF, Best JH. A network meta-analysis to compare glycaemic control in patients with type 2 diabetes treated with exenatide once weekly or liraglutide once daily in comparison with insulin glargine, exenatide twice daily or placebo. Diabetes Obes Metab. 2013;15(3):213-23.

281. Shamliyan TA, Choi JY, Ramakrishnan R, Miller JB, Wang SY, Taylor FR, et al. Preventive pharmacologic treatments for episodic migraine in adults. J Gen Intern Med. 2013;28(9):1225-37.

282. Shamliyan TA, Kane RL, Taylor FR. AHRQ Comparative Effectiveness Reviews. Migraine in Adults: Preventive Pharmacologic Treatments. Rockville (MD): Agency for Healthcare Research and Quality (US); 2013.

283. Shi KQ, Liu WY, Pan ZZ, Ling XF, Chen SL, Chen YP, et al. Secondary prophylaxis of variceal bleeding for cirrhotic patients: a multiple-treatments meta-analysis. Eur J Clin Invest. 2013;43(8):844-54.

284. Shu T, Chen GH, Rong L, Feng F, Yang B, Chen R, et al. Indirect comparison of anti-TNF-alpha agents for active ankylosing spondylitis: mixed treatment comparison of randomized controlled trials. Clin Exp Rheumatol. 2013;31(5):717-22.

285. Skoetz N, Trelle S, Rancea M, Haverkamp H, Diehl V, Engert A, et al. Effect of initial treatment strategy on survival of patients with advanced-stage Hodgkin's lymphoma: a systematic review and network meta-analysis. Lancet Oncol. 2013;14(10):943-52.

286. Terasawa T, Trikalinos NA, Djulbegovic B, Trikalinos TA. Comparative efficacy of first-line therapies for advanced-stage chronic lymphocytic leukemia: a multiple-treatment meta-analysis. Cancer Treat Rev. 2013;39(4):340-9.

287. Uthman OA, van der Windt DA, Jordan JL, Dziedzic KS, Healey EL, Peat GM, et al. Exercise for lower limb osteoarthritis: systematic review incorporating trial sequential analysis and network meta-analysis. BMJ. 2013;347:f5555.

288. Wiens A, Lenzi L, Venson R, Correr CJ, Rotta I, Pedroso ML, et al. Comparative efficacy of oral nucleoside or nucleotide analog monotherapy used in chronic hepatitis B: a mixed-treatment comparison meta-analysis. Pharmacotherapy. 2013;33(2):144-51.

289. Wu HY, Huang JW, Lin HJ, Liao WC, Peng YS, Hung KY, et al. Comparative effectiveness of renin-angiotensin system blockers and other antihypertensive drugs in patients with diabetes: systematic review and bayesian network meta-analysis. BMJ. 2013;347:f6008.

290. Wu MS, Tan SC, Xiong T. Indirect comparison of randomised controlled trials: comparative efficacy of dexlansoprazole vs. esomeprazole in the treatment of gastro-oesophageal reflux disease. Aliment Pharmacol Ther. 2013;38(2):190-201.

291. Yuan J, Zhang R, Yang Z, Lee J, Liu Y, Tian J, et al. Comparative effectiveness and safety of oral phosphodiesterase type 5 inhibitors for erectile dysfunction: a systematic review and network meta-analysis. Eur Urol. 2013;63(5):902-12.

292. Zaccara G, Giovannelli F, Maratea D, Fadda V, Verrotti A. Neurological adverse events of new generation sodium blocker antiepileptic drugs. Meta-analysis of randomized, double-blinded studies with eslicarbazepine acetate, lacosamide and oxcarbazepine. Seizure. 2013;22(7):528-36.

293. Zhang HNS, R. A network meta-analysis on the influence of antihypertensive agents on new-onset diabetes [Chinese]. Chinese Pharmaceutical Journal. 2013;48(11):930-4.

294. Ashby RL, Gabe R, Ali S, Saramago P, Chuang LH, Adderley U, et al. VenUS IV (Venous leg Ulcer Study IV) - compression hosiery compared with compression bandaging in the treatment of venous leg ulcers: a randomised controlled trial, mixed-treatment comparison and decision-analytic model. Health Technol Assess. 2014;18(57):1-293, v-vi.

295. Baji P, Pentek M, Czirjak L, Szekanecz Z, Nagy G, Gulacsi L, et al. Efficacy and safety of infliximab-biosimilar compared to other biological drugs in rheumatoid arthritis: a mixed treatment comparison. Eur J Health Econ. 2014;15 Suppl 1:S53-64.

296. Baji P, Pentek M, Szanto S, Geher P, Gulacsi L, Balogh O, et al. Comparative efficacy and safety of biosimilar infliximab and other biological treatments in ankylosing spondylitis: systematic literature review and meta-analysis. Eur J Health Econ. 2014;15 Suppl 1:S45-52.

297. Bangalore S, Toklu B, Feit F. Outcomes with coronary artery bypass graft surgery versus percutaneous coronary intervention for patients with diabetes mellitus: can newer generation drug-eluting stents bridge the gap? Circ Cardiovasc Interv. 2014;7(4):518-25.

298. Bangalore S, Toklu B, Kotwal A, Volodarskiy A, Sharma S, Kirtane AJ, et al. Anticoagulant therapy during primary percutaneous coronary intervention for acute myocardial infarction: a meta-analysis of randomized trials in the era of stents and P2Y12 inhibitors. BMJ. 2014;349:g6419.

299. Biondi-Zoccai G, Lotrionte M, Thomsen HS, Romagnoli E, D'Ascenzo F, Giordano A, et al. Nephropathy after administration of iso-osmolar and low-osmolar contrast media: evidence from a network meta-analysis. Int J Cardiol. 2014;172(2):375-80.

300. Caldeira D, Pinto FJ, Ferreira JJ. Dyspnea and reversibility profile of P2Y(1)(2) antagonists: systematic review of new antiplatelet drugs. Am J Cardiovasc Drugs. 2014;14(4):303-11.

301. Cameron C, Coyle D, Richter T, Kelly S, Gauthier K, Steiner S, et al. Systematic review and network meta-analysis comparing antithrombotic agents for the prevention of stroke and major bleeding in patients with atrial fibrillation. BMJ Open. 2014;4(6):e004301.

302. Carter P, Achana F, Troughton J, Gray LJ, Khunti K, Davies MJ. A Mediterranean diet improves HbA1c but not fasting blood glucose compared to alternative dietary strategies: a network meta-analysis. J Hum Nutr Diet. 2014;27(3):280-97.

303. Caruba T, Katsahian S, Schramm C, Charles Nelson A, Durieux P, Begue D, et al. Treatment for stable coronary artery disease: a network meta-analysis of cost-effectiveness studies. PLoS One. 2014;9(6):e98371.

304. Castellucci LA, Cameron C, Le Gal G, Rodger MA, Coyle D, Wells PS, et al. Clinical and safety outcomes associated with treatment of acute venous thromboembolism: a systematic review and meta-analysis. JAMA. 2014;312(11):1122-35.

305. Chambers JD, Winn A, Zhong Y, Olchanski N, Cangelosi MJ. Potential role of network meta-analysis in value-based insurance design. Am J Manag Care. 2014;20(8):641-8.

306. Chan K, Shah K, Lien K, Coyle D, Lam H, Ko YJ. A Bayesian meta-analysis of multiple treatment comparisons of systemic regimens for advanced pancreatic cancer. PLoS One. 2014;9(10):e108749.

307. Chen Y, Zhang Y, Tang Y, Huang X, Xie Y. Long-term clinical efficacy and safety of adding cilostazol to dual antiplatelet therapy for patients undergoing PCI: a meta-analysis of randomized trials with adjusted indirect comparisons. Curr Med Res Opin. 2014;30(1):37-49.

308. Chilton M, Dunkley A, Carter P, Davies MJ, Khunti K, Gray LJ. The effect of antiobesity drugs on waist circumference: a mixed treatment comparison. Diabetes Obes Metab. 2014;16(3):237-47.

309. Craddy P, Palin HJ, Johnson KI. Comparative effectiveness of dipeptidylpeptidase-4 inhibitors in type 2 diabetes: a systematic review and mixed treatment comparison. Diabetes Ther. 2014;5(1):1-41.

310. Cucherat M, Stalmans I, Rouland JF. Relative efficacy and safety of preservative-free latanoprost (T2345) for the treatment of open-angle glaucoma and ocular hypertension: an adjusted Indirect comparison meta-analysis of randomized clinical trials. J Glaucoma. 2014;23(1):e69-75.

311. Cui J, Wu B, Liu C, Li Z. A systematic review and adjusted indirect comparison of oral anticoagulants. Orthopedics. 2014;37(11):763-71.

312. Dai N, Xu D, Zhang J, Wei Y, Li W, Fan B, et al. Different beta-blockers and initiation time in patients undergoing noncardiac surgery: a meta-analysis. Am J Med Sci. 2014;347(3):235-44.

313. Dai X, Wang H, Jing Z, Fu P. The effect of a dual combination of noninsulin antidiabetic drugs on lipids: a systematic review and network meta-analysis. Curr Med Res Opin. 2014;30(9):1777-86.

314. Dal Molin A, Allara E, Montani D, Milani S, Frassati C, Cossu S, et al. Flushing the central venous catheter: is heparin necessary? J Vasc Access. 2014;15(4):241-8.

315. Desamericq G, Schurhoff F, Meary A, Szoke A, Macquin-Mavier I, Bachoud-Levi AC, et al. Long-term neurocognitive effects of antipsychotics in schizophrenia: a network meta-analysis. Eur J Clin Pharmacol. 2014;70(2):127-34.

316. Dogliotti A, Paolasso E, Giugliano RP. Current and new oral antithrombotics in non-valvular atrial fibrillation: a network meta-analysis of 79 808 patients. Heart. 2014;100(5):396-405.

317. Dong J, Gao L, Lu W, Xu Z, Zheng J. Pharmacological interventions for acceleration of the onset time of rocuronium: a meta-analysis. PLoS One. 2014;9(12):e114231.

318. Dooley C, Kaur R, Sobieraj DM. Comparison of the efficacy and safety of low molecular weight heparins for venous thromboembolism prophylaxis in medically ill patients. Curr Med Res Opin. 2014;30(3):367-80.

319. Dranitsaris G, Ellis AK. Sublingual or subcutaneous immunotherapy for seasonal allergic rhinitis: an indirect analysis of efficacy, safety and cost. J Eval Clin Pract. 2014;20(3):225-38.

320. Ellis AG, Reginster JY, Luo X, Bushmakin AG, Williams R, Sutradhar S, et al. Indirect comparison of bazedoxifene vs oral bisphosphonates for the prevention of vertebral fractures in postmenopausal osteoporotic women. Curr Med Res Opin. 2014;30(8):1617-26.

321. Ellis AG, Reginster JY, Luo X, Cappelleri JC, Chines A, Sutradhar S, et al. Bazedoxifene versus oral bisphosphonates for the prevention of nonvertebral fractures in postmenopausal women with osteoporosis at higher risk of fracture: a network meta-analysis. Value Health. 2014;17(4):424-32.

322. Faggion CM, Jr., Listl S, Fruhauf N, Chang HJ, Tu YK. A systematic review and Bayesian network meta-analysis of randomized clinical trials on non-surgical treatments for peri-implantitis. J Clin Periodontol. 2014;41(10):1015-25.

323. Fournier M, Germe M, Theobald K, Scholz GH, Lehmacher W. Indirect comparison of lixisenatide versus neutral protamine Hagedorn insulin as add-on to metformin and sulphonylurea in patients with type 2 diabetes mellitus. Ger Med Sci. 2014;12:Doc14.

324. Furukawa TA, Noma H, Caldwell DM, Honyashiki M, Shinohara K, Imai H, et al. Waiting list may be a nocebo condition in psychotherapy trials: a contribution from network meta-analysis. Acta Psychiatr Scand. 2014;130(3):181-92.

325. Gerger H, Munder T, Gemperli A, Nuesch E, Trelle S, Juni P, et al. Integrating fragmented evidence by network meta-analysis: relative effectiveness of psychological interventions for adults with post-traumatic stress disorder. Psychol Med. 2014;44(15):3151-64.

326. Gonzalez-Vacarezza N, Aleman A, Gonzalez G, Perez A. Rituximab and tocilizumab for the treatment of rheumatoid arthritis. Int J Technol Assess Health Care. 2014;30(3):282-8.

327. Goring S, Hawkins N, Wygant G, Roudaut M, Townsend R, Wood I, et al. Dapagliflozin compared with other oral anti-diabetes treatments when added to metformin monotherapy: a systematic review and network meta-analysis. Diabetes Obes Metab. 2014;16(5):433-42.

328. Graudal N, Hubeck-Graudal T, Tarp S, Christensen R, Jurgens G. Effect of combination therapy on joint destruction in rheumatoid arthritis: a network meta-analysis of randomized controlled trials. PLoS One. 2014;9(9):e106408.

329. Gresham GK, Wells GA, Gill S, Cameron C, Jonker DJ. Chemotherapy regimens for advanced pancreatic cancer: a systematic review and network meta-analysis. BMC Cancer. 2014;14:471.

330. Griebeler ML, Morey-Vargas OL, Brito JP, Tsapas A, Wang Z, Leon BGC, et al. Pharmacologic interventions for painful diabetic neuropathy: an umbrella systematic review and comparative effectiveness network meta-analysis. Ann Intern Med. 2014;161(9):639-49.

331. Gupta AK, Charrette A. The efficacy and safety of 5alpha-reductase inhibitors in androgenetic alopecia: a network meta-analysis and benefit-risk assessment of finasteride and dutasteride. J Dermatolog Treat. 2014;25(2):156-61.

332. Gupta AK, Daigle D, Lyons DC. Network Meta-analysis of Treatments for Chronic Plaque Psoriasis in Canada. J Cutan Med Surg. 2014;18(6):371-8.

333. Hornyak M, Scholz H, Kohnen R, Bengel J, Kassubek J, Trenkwalder C. What treatment works best for restless legs syndrome? Meta-analyses of dopaminergic and non-dopaminergic medications. Sleep Med Rev. 2014;18(2):153-64.

334. Hutchinson M, Fox RJ, Havrdova E, Kurukulasuriya NC, Sarda SP, Agarwal S, et al. Efficacy and safety of BG-12 (dimethyl fumarate) and other disease-modifying therapies for the treatment of relapsing-remitting multiple sclerosis: a systematic review and mixed treatment comparison. Curr Med Res Opin. 2014;30(4):613-27.

335. Jansen JP, Buckley F, Dejonckheere F, Ogale S. Comparative efficacy of biologics as monotherapy and in combination with methotrexate on patient reported outcomes (PROs) in rheumatoid arthritis patients with an inadequate response to conventional DMARDs--a systematic review and network meta-analysis. Health Qual Life Outcomes. 2014;12:102.

336. Johnston BC, Kanters S, Bandayrel K, Wu P, Naji F, Siemieniuk RA, et al. Comparison of weight loss among named diet programs in overweight and obese adults: a meta-analysis. JAMA. 2014;312(9):923-33.

337. Kang N, Sobieraj DM. Indirect treatment comparison of new oral anticoagulants for the treatment of acute venous thromboembolism. Thromb Res. 2014;133(6):1145-51.

338. Katsanos K, Spiliopoulos S, Karunanithy N, Krokidis M, Sabharwal T, Taylor P. Bayesian network meta-analysis of nitinol stents, covered stents, drug-eluting stents, and drug-coated balloons in the femoropopliteal artery. J Vasc Surg. 2014;59(4):1123-33.e8.

339. Kew KM, Dias S, Cates CJ. Long-acting inhaled therapy (beta-agonists, anticholinergics and steroids) for COPD: a network meta-analysis. Cochrane Database Syst Rev. 2014;3:Cd010844.

340. Kriston L, von Wolff A, Westphal A, Holzel LP, Harter M. Efficacy and acceptability of acute treatments for persistent depressive disorder: a network meta-analysis. Depress Anxiety. 2014;31(8):621-30.

341. Kunitomi T, Hashiguchi M, Mochizuki M. Indirect comparison analysis of efficacy and safety between olanzapine and aripiprazole for schizophrenia. Br J Clin Pharmacol. 2014;77(5):767-76.

342. Laporte S, Chapelle C, Bertoletti L, Lega JC, Cucherat M, Zufferey PJ, et al. Indirect comparison meta-analysis of two enoxaparin regimens in patients undergoing major orthopaedic surgery. Impact on the interpretation of thromboprophylactic effects of new anticoagulant drugs. Thromb Haemost. 2014;112(3):503-10.

343. Le Cleach L, Trinquart L, Do G, Maruani A, Lebrun-Vignes B, Ravaud P, et al. Oral antiviral therapy for prevention of genital herpes outbreaks in immunocompetent and nonpregnant patients. Cochrane Database Syst Rev. 2014;8:Cd009036.

344. Leaviss J, Sullivan W, Ren S, Everson-Hock E, Stevenson M, Stevens JW, et al. What is the clinical effectiveness and cost-effectiveness of cytisine compared with varenicline for smoking cessation? A systematic review and economic evaluation. Health Technol Assess. 2014;18(33):1-120.

345. Leibovici-Weissman Y, Neuberger A, Bitterman R, Sinclair D, Salam MA, Paul M. Antimicrobial drugs for treating cholera. Cochrane Database Syst Rev. 2014;6:Cd008625.

346. Li L, Tian J, Tian H, Sun R, Wang Q, Yang K. The efficacy and safety of different kinds of laparoscopic cholecystectomy: a network meta analysis of 43 randomized controlled trials. PLoS One. 2014;9(2):e90313.

347. Liang W, Wu X, Fang W, Zhao Y, Yang Y, Hu Z, et al. Network meta-analysis of erlotinib, gefitinib, afatinib and icotinib in patients with advanced non-small-cell lung cancer harboring EGFR mutations. PLoS One. 2014;9(2):e85245.

348. Lin L, Zhao YJ, Chew PT, Sng CC, Wong HT, Yip LW, et al. Comparative efficacy and tolerability of topical prostaglandin analogues for primary open-angle glaucoma and ocular hypertension. Ann Pharmacother. 2014;48(12):1585-93.

349. Lin PY, Chen HS, Wang YH, Tu YK. Primary molar pulpotomy: a systematic review and network meta-analysis. J Dent. 2014;42(9):1060-77.

350. Liu Y, Yan R, Song A, Niu X, Cao C, Wei J, et al. Aliskiren/amlodipine vs. aliskiren/hydrochlorothiazide in hypertension: indirect meta-analysis of trials comparing the two combinations vs. monotherapy. Am J Hypertens. 2014;27(2):268-78.

351. Llorca PM, Lancon C, Brignone M, Rive B, Salah S, Ereshefsky L, et al. Relative efficacy and tolerability of vortioxetine versus selected antidepressants by indirect comparisons of similar clinical studies. Curr Med Res Opin. 2014;30(12):2589-606.

352. Loke YK, Pradhan S, Yeong JK, Kwok CS. Comparative coronary risks of apixaban, rivaroxaban and dabigatran: a meta-analysis and adjusted indirect comparison. Br J Clin Pharmacol. 2014;78(4):707-17.

353. Loveman E, Copley VR, Colquitt JL, Scott DA, Clegg AJ, Jones J, et al. The effectiveness and cost-effectiveness of treatments for idiopathic pulmonary fibrosis: systematic review, network meta-analysis and health economic evaluation. BMC Pharmacol Toxicol. 2014;15:63.

354. Loymans RJ, Gemperli A, Cohen J, Rubinstein SM, Sterk PJ, Reddel HK, et al. Comparative effectiveness of long term drug treatment strategies to prevent asthma exacerbations: network meta-analysis. BMJ. 2014;348:g3009.

355. Maman K, Aballea S, Nazir J, Desroziers K, Neine ME, Siddiqui E, et al. Comparative efficacy and safety of medical treatments for the management of overactive bladder: a systematic literature review and mixed treatment comparison. Eur Urol. 2014;65(4):755-65.

356. Mealing S, Ghement I, Hawkins N, Scott DA, Lescrauwaet B, Watt M, et al. The importance of baseline viral load when assessing relative efficacy in treatment-naive HBeAg-positive chronic hepatitis B: a systematic review and network meta-analysis. Syst Rev. 2014;3:21.

357. Messori A, Fadda V, Maratea D, Trippoli S, Gatto R, De Rosa M, et al. Biological drugs for the treatment of rheumatoid arthritis by the subcutaneous route: interpreting efficacy data to assess statistical equivalence. Ther Adv Musculoskelet Dis. 2014;6(6):207-16.

358. Messori A, Fadda V, Maratea D, Trippoli S, Gatto R, De Rosa M, et al. Biological drugs for the treatment of moderate-to-severe psoriasis by subcutaneous route: determining statistical equivalence according to evidence-based methods. Clin Drug Investig. 2014;34(8):593-8.

359. Messori A, Fadda V, Maratea D, Trippoli S, Marinai C. Anti-reabsorptive agents in women with osteoporosis: determining statistical equivalence according to evidence-based methods. J Endocrinol Invest. 2014;37(8):769-73.

360. Mills EJ, Lester R, Thorlund K, Lorenzi M, Muldoon K, Kanters S, et al. Interventions to promote adherence to antiretroviral therapy in Africa: a network meta-analysis. The Lancet HIV. 2014;1(3):e104-e11.

361. Mills EJ, Thorlund K, Eapen S, Wu P, Prochaska JJ. Cardiovascular events associated with smoking cessation pharmacotherapies: a network meta-analysis. Circulation. 2014;129(1):28-41.

362. Musini VM, Nazer M, Bassett K, Wright JM. Blood pressure-lowering efficacy of monotherapy with thiazide diuretics for primary hypertension. Cochrane Database Syst Rev. 2014;5:Cd003824.

363. Myers J, Wielage RC, Han B, Price K, Gahn J, Paget MA, et al. The efficacy of duloxetine, non-steroidal anti-inflammatory drugs, and opioids in osteoarthritis: a systematic literature review and meta-analysis. BMC Musculoskelet Disord. 2014;15:76.

364. Naci H, Dias S, Ades AE. Industry sponsorship bias in research findings: a network meta-analysis of LDL cholesterol reduction in randomised trials of statins. BMJ. 2014;349:g5741.

365. Nagayama A, Hayashida T, Jinno H, Takahashi M, Seki T, Matsumoto A, et al. Comparative effectiveness of neoadjuvant therapy for HER2-positive breast cancer: a network meta-analysis. J Natl Cancer Inst. 2014;106(9).

366. Oba Y, Lone NA. Comparative efficacy of inhaled corticosteroid and long-acting beta agonist combinations in preventing COPD exacerbations: a Bayesian network meta-analysis. Int J Chron Obstruct Pulmon Dis. 2014;9:469-79.

367. Oba Y, Lone NA. Mortality benefit of vasopressor and inotropic agents in septic shock: a Bayesian network meta-analysis of randomized controlled trials. J Crit Care. 2014;29(5):706-10.

368. Orme M, Fenici P, Lomon ID, Wygant G, Townsend R, Roudaut M. A systematic review and mixed-treatment comparison of dapagliflozin with existing anti-diabetes treatments for those with type 2 diabetes mellitus inadequately controlled by sulfonylurea monotherapy. Diabetol Metab Syndr. 2014;6:73.

369. Palmer SC, Saglimbene V, Mavridis D, Salanti G, Craig JC, Tonelli M, et al. Erythropoiesis-stimulating agents for anaemia in adults with chronic kidney disease: a network meta-analysis. Cochrane Database Syst Rev. 2014;12:Cd010590.

370. Palmerini T, Biondi-Zoccai G, Della Riva D, Mariani A, Sabate M, Smits PC, et al. Clinical outcomes with bioabsorbable polymer- versus durable polymer-based drug-eluting and bare-metal stents: evidence from a comprehensive network meta-analysis. J Am Coll Cardiol. 2014;63(4):299-307.

371. Patel DA, Snedecor SJ, Tang WY, Sudharshan L, Lim JW, Cuffe R, et al. 48-week efficacy and safety of dolutegravir relative to commonly used third agents in treatment-naive HIV-1-infected patients: a systematic review and network meta-analysis. PLoS One. 2014;9(9):e105653.

372. Pechlivanoglou P, Le HH, Daenen S, Snowden JA, Postma MJ. Mixed treatment comparison of prophylaxis against invasive fungal infections in neutropenic patients receiving therapy for haematological malignancies: a systematic review. J Antimicrob Chemother. 2014;69(1):1-11.

373. Peruzzi M, De Luca L, Thomsen HS, Romagnoli E, D'Ascenzo F, Mancone M, et al. A network meta-analysis on randomized trials focusing on the preventive effect of statins on contrast-induced nephropathy. Biomed Res Int. 2014;2014:213239.

374. Piccolo R, Galasso G, Piscione F, Esposito G, Trimarco B, Dangas GD, et al. Meta-analysis of randomized trials comparing the effectiveness of different strategies for the treatment of drug-eluting stent restenosis. Am J Cardiol. 2014;114(9):1339-46.

375. Price R, MacLennan G, Glen J. Selective digestive or oropharyngeal decontamination and topical oropharyngeal chlorhexidine for prevention of death in general intensive care: systematic review and network meta-analysis. BMJ. 2014;348:g2197.

376. Regnier S, Malcolm W, Allen F, Wright J, Bezlyak V. Efficacy of anti-VEGF and laser photocoagulation in the treatment of visual impairment due to diabetic macular edema: a systematic review and network meta-analysis. PLoS One. 2014;9(7):e102309.

377. Rochwerg B, Alhazzani W, Sindi A, Heels-Ansdell D, Thabane L, Fox-Robichaud A, et al. Fluid resuscitation in sepsis: a systematic review and network meta-analysis. Ann Intern Med. 2014;161(5):347-55.

378. Rollins BM, Silva MA, Donovan JL, Kanaan AO. Evaluation of oral anticoagulants for the extended treatment of venous thromboembolism using a mixed-treatment comparison, meta-analytic approach. Clin Ther. 2014;36(10):1454-64.e3.

379. Roskell NS, Anzueto A, Hamilton A, Disse B, Becker K. Once-daily long-acting beta-agonists for chronic obstructive pulmonary disease: an indirect comparison of olodaterol and indacaterol. Int J Chron Obstruct Pulmon Dis. 2014;9:813-24.

380. Roskell NS, Setyawan J, Zimovetz EA, Hodgkins P. Systematic evidence synthesis of treatments for ADHD in children and adolescents: indirect treatment comparisons of lisdexamfetamine with methylphenidate and atomoxetine. Curr Med Res Opin. 2014;30(8):1673-85.

381. Schmitt J, Rosumeck S, Thomaschewski G, Sporbeck B, Haufe E, Nast A. Efficacy and safety of systemic treatments for moderate-to-severe psoriasis: meta-analysis of randomized controlled trials. Br J Dermatol. 2014;170(2):274-303.

382. Shams T, Firwana B, Habib F, Alshahrani A, Alnouh B, Murad MH, et al. SSRIs for hot flashes: a systematic review and meta-analysis of randomized trials. J Gen Intern Med. 2014;29(1):204-13.

383. Singh S, Garg SK, Pardi DS, Wang Z, Murad MH, Loftus EV, Jr. Comparative efficacy of biologic therapy in biologic-naive patients with Crohn disease: a systematic review and network meta-analysis. Mayo Clin Proc. 2014;89(12):1621-35.

384. Snedecor SJ, Sudharshan L, Cappelleri JC, Sadosky A, Desai P, Jalundhwala Y, et al. Systematic review and meta-analysis of pharmacological therapies for pain associated with postherpetic neuralgia and less common neuropathic conditions. Int J Clin Pract. 2014;68(7):900-18.

385. Snedecor SJ, Sudharshan L, Cappelleri JC, Sadosky A, Mehta S, Botteman M. Systematic review and meta-analysis of pharmacological therapies for painful diabetic peripheral neuropathy. Pain Pract. 2014;14(2):167-84.

386. Stagg HR, Zenner D, Harris RJ, Munoz L, Lipman MC, Abubakar I. Treatment of latent tuberculosis infection: a network meta-analysis. Ann Intern Med. 2014;161(6):419-28.

387. Stidham RW, Lee TC, Higgins PD, Deshpande AR, Sussman DA, Singal AG, et al. Systematic review with network meta-analysis: the efficacy of anti-TNF agents for the treatment of Crohn's disease. Aliment Pharmacol Ther. 2014;39(12):1349-62.

388. Stidham RW, Lee TC, Higgins PD, Deshpande AR, Sussman DA, Singal AG, et al. Systematic review with network meta-analysis: the efficacy of anti-tumour necrosis factor-alpha agents for the treatment of ulcerative colitis. Aliment Pharmacol Ther. 2014;39(7):660-71.

389. Sun Y, van Valkenhoef G, Morel T. A mixed treatment comparison of gabapentin enacarbil, pramipexole, ropinirole and rotigotine in moderate-to-severe restless legs syndrome. Curr Med Res Opin. 2014;30(11):2267-78.

390. Tadrous M, Wong L, Mamdani MM, Juurlink DN, Krahn MD, Levesque LE, et al. Comparative gastrointestinal safety of bisphosphonates in primary osteoporosis: a network meta-analysis. Osteoporos Int. 2014;25(4):1225-35.

391. Thorlund K, Mills EJ, Wu P, Ramos E, Chatterjee A, Druyts E, et al. Comparative efficacy of triptans for the abortive treatment of migraine: a multiple treatment comparison meta-analysis. Cephalalgia. 2014;34(4):258-67.

392. Thorlund K, Wu P, Druyts E, Eapen S, Mills EJ. Nonergot dopamine-receptor agonists for treating Parkinson's disease - a network meta-analysis. Neuropsychiatr Dis Treat. 2014;10:767-76.

393. Vegter S, Tolley K. A network meta-analysis of the relative efficacy of treatments for actinic keratosis of the face or scalp in Europe. PLoS One. 2014;9(6):e96829.

394. Wang H, Yuan J, Hu X, Tao K, Liu J, Hu D. The effectiveness and safety of avanafil for erectile dysfunction: a systematic review and meta-analysis. Curr Med Res Opin. 2014;30(8):1565-71.

395. Wang JC, Tian JH, Ge L, Gan YH, Yang KH. Which is the best Chinese herb injection based on the FOLFOX regimen for gastric cancer? A network meta- analysis of randomized controlled trials. Asian Pac J Cancer Prev. 2014;15(12):4795-800.

396. Wang X, Wang X, Li S, Meng Z, Liu T, Zhang X. Comparative effectiveness of oral drug therapies for lower urinary tract symptoms due to benign prostatic hyperplasia: a systematic review and network meta-analysis. PLoS One. 2014;9(9):e107593.

397. Wertli MM, Kessels AG, Perez RS, Bachmann LM, Brunner F. Rational pain management in complex regional pain syndrome 1 (CRPS 1)--a network meta-analysis. Pain Med. 2014;15(9):1575-89.

398. Windecker S, Stortecky S, Stefanini GG, da Costa BR, Rutjes AW, Di Nisio M, et al. Revascularisation versus medical treatment in patients with stable coronary artery disease: network meta-analysis. BMJ. 2014;348:g3859.

399. Xiong T, Turner RM, Wei Y, Neal DE, Lyratzopoulos G, Higgins JP. Comparative efficacy and safety of treatments for localised prostate cancer: an application of network meta-analysis. BMJ Open. 2014;4(5):e004285.

400. Yang B, Shi J, Chen X, Ma B, Sun H. Efficacy and safety of therapies for acute ischemic stroke in China: a network meta-analysis of 13289 patients from 145 randomized controlled trials. PLoS One. 2014;9(2):e88440.

401. Yang Z, Ye X, Wu Q, Wu K, Fan D. A network meta-analysis on the efficacy of 5-aminosalicylates, immunomodulators and biologics for the prevention of postoperative recurrence in Crohn's disease. Int J Surg. 2014;12(5):516-22.

402. Zeppetella G, Davies A, Eijgelshoven I, Jansen JP. A network meta-analysis of the efficacy of opioid analgesics for the management of breakthrough cancer pain episodes. J Pain Symptom Manage. 2014;47(4):772-85.e5.

403. Zhang YW, Zhang YL, Pan H, Wei FX, Zhang YC, Shao Y, et al. Chemotherapy for patients with gastric cancer after complete resection: a network meta-analysis. World J Gastroenterol. 2014;20(2):584-92.

404. Zhu Z, Zhang J, Liu Y, Chen M, Guo P, Li K. Efficacy and toxicity of external-beam radiation therapy for localised prostate cancer: a network meta-analysis. Br J Cancer. 2014;110(10):2396-404.

405. Ziakas PD, Zervou FN, Zacharioudakis IM, Mylonakis E. Graft-versus-host disease prophylaxis after transplantation: a network meta-analysis. PLoS One. 2014;9(12):e114735.

406. Zintzaras E, Miligkos M, Ziakas P, Balk EM, Mademtzoglou D, Doxani C, et al. Assessment of the relative effectiveness and tolerability of treatments of type 2 diabetes mellitus: a network meta-analysis. Clin Ther. 2014;36(10):1443-53.e9.

407. Alfirevic Z, Keeney E, Dowswell T, Welton NJ, Dias S, Jones LV, et al. Labour induction with prostaglandins: a systematic review and network meta-analysis. BMJ. 2015;350:h217.

408. Bannuru RR, Schmid CH, Kent DM, Vaysbrot EE, Wong JB, McAlindon TE. Comparative effectiveness of pharmacologic interventions for knee osteoarthritis: a systematic review and network meta-analysis. Ann Intern Med. 2015;162(1):46-54.

409. Benedetto U, Raja SG, Albanese A, Amrani M, Biondi-Zoccai G, Frati G. Searching for the second best graft for coronary artery bypass surgery: a network meta-analysis of randomized controlled trialsdagger. Eur J Cardiothorac Surg. 2015;47(1):59-65; discussion

410. Bow EJ, Vanness DJ, Slavin M, Cordonnier C, Cornely OA, Marks DI, et al. Systematic review and mixed treatment comparison meta-analysis of randomized clinical trials of primary oral antifungal prophylaxis in allogeneic hematopoietic cell transplant recipients. BMC Infect Dis. 2015;15:128.

411. Bulluck H, Kwok CS, Ryding AD, Loke YK. Safety of short-term dual antiplatelet therapy after drug-eluting stents: An updated meta-analysis with direct and adjusted indirect comparison of randomized control trials. Int J Cardiol. 2015;181:331-9.

412. Chen L, Staubli SE, Schneider MP, Kessels AG, Ivic S, Bachmann LM, et al. Phosphodiesterase 5 inhibitors for the treatment of erectile dysfunction: a trade-off network meta-analysis. Eur Urol. 2015;68(4):674-80.

413. Dong W, Goost H, Lin XB, Burger C, Paul C, Wang ZL, et al. Treatments for shoulder impingement syndrome: a PRISMA systematic review and network meta-analysis. Medicine (Baltimore). 2015;94(10):e510.

414. Edwards SJ, Barton S, Thurgar E, Trevor N. Topotecan, pegylated liposomal doxorubicin hydrochloride, paclitaxel, trabectedin and gemcitabine for advanced recurrent or refractory ovarian cancer: a systematic review and economic evaluation. Health Technol Assess. 2015;19(7):1-480.

415. Galvan-Banqueri M, Vega-Coca MD, Castillo-Munoz MA, Beltran Calvo C, Molina Lopez T. Indirect comparison for Anti-TNF drugs in moderate to severe ulcerative colitis. Farm Hosp. 2015;39(2):80-91.

416. Greco T, Calabro MG, Covello RD, Greco M, Pasin L, Morelli A, et al. A Bayesian network meta-analysis on the effect of inodilatory agents on mortality. Br J Anaesth. 2015;114(5):746-56.

417. Haspinger ER, Agustoni F, Torri V, Gelsomino F, Platania M, Zilembo N, et al. Is there evidence for different effects among EGFR-TKIs? Systematic review and meta-analysis of EGFR tyrosine kinase inhibitors (TKIs) versus chemotherapy as first-line treatment for patients harboring EGFR mutations. Crit Rev Oncol Hematol. 2015;94(2):213-27.

418. Hazlewood GS, Rezaie A, Borman M, Panaccione R, Ghosh S, Seow CH, et al. Comparative effectiveness of immunosuppressants and biologics for inducing and maintaining remission in Crohn's disease: a network meta-analysis. Gastroenterology. 2015;148(2):344-54.e5; quiz e14-5.

419. Huai ZY, Feng Xian W, Chang Jiang L, Xi Chen W. Submucosal injection solution for endoscopic resection in gastrointestinal tract: a traditional and network meta-analysis. Gastroenterol Res Pract. 2015;2015:702768.

420. Huttner FJ, Tenckhoff S, Jensen K, Uhlmann L, Kulu Y, Buchler MW, et al. Meta-analysis of reconstruction techniques after low anterior resection for rectal cancer. Br J Surg. 2015;102(7):735-45.

421. Kotb A, Cameron C, Hsieh S, Wells G. Comparative effectiveness of different forms of telemedicine for individuals with heart failure (HF): a systematic review and network meta-analysis. PLoS One. 2015;10(2):e0118681.

422. Kunitomi T, Hashiguchi M, Mochizuki M. Effect of common comparators in indirect comparison analysis of the effectiveness of different inhaled corticosteroids in the treatment of asthma. PLoS One. 2015;10(3):e0120836.

423. Lee SW, Chaiyakunapruk N, Chong HY, Liong ML. Comparative effectiveness and safety of various treatment procedures for lower pole renal calculi: a systematic review and network meta-analysis. BJU Int. 2015;116(2):252-64.

424. Lewis RA, Williams NH, Sutton AJ, Burton K, Din NU, Matar HE, et al. Comparative clinical effectiveness of management strategies for sciatica: systematic review and network meta-analyses. Spine J. 2015;15(6):1461-77.

425. Linde K, Kriston L, Rucker G, Jamil S, Schumann I, Meissner K, et al. Efficacy and acceptability of pharmacological treatments for depressive disorders in primary care: systematic review and network meta-analysis. Ann Fam Med. 2015;13(1):69-79.

426. Loveman E, Copley VR, Colquitt J, Scott DA, Clegg A, Jones J, et al. The clinical effectiveness and cost-effectiveness of treatments for idiopathic pulmonary fibrosis: a systematic review and economic evaluation. Health Technol Assess. 2015;19(20):i-xxiv, 1-336.

427. Mantha S, Ansell J. Indirect comparison of dabigatran, rivaroxaban, apixaban and edoxaban for the treatment of acute venous thromboembolism. J Thromb Thrombolysis. 2015;39(2):155-65.

428. Maxwell LJ, Zochling J, Boonen A, Singh JA, Veras MM, Tanjong Ghogomu E, et al. TNF-alpha inhibitors for ankylosing spondylitis. Cochrane Database Syst Rev. 2015;4:Cd005468.

429. Messori A, Fadda V, Maratea D, Trippoli S. First-line treatments for chronic lymphocytic leukaemia: interpreting efficacy data by network meta-analysis. Ann Hematol. 2015;94(6):1003-9.

430. Moja L, Danese S, Fiorino G, Del Giovane C, Bonovas S. Systematic review with network meta-analysis: comparative efficacy and safety of budesonide and mesalazine (mesalamine) for Crohn's disease. Aliment Pharmacol Ther. 2015;41(11):1055-65.

431. Nelson H, Cartier S, Allen-Ramey F, Lawton S, Calderon MA. Network meta-analysis shows commercialized subcutaneous and sublingual grass products have comparable efficacy. J Allergy Clin Immunol Pract. 2015;3(2):256-66.e3.

432. Oba Y, Lone NA. Comparative efficacy of long-acting muscarinic antagonists in preventing COPD exacerbations: a network meta-analysis and meta-regression. Ther Adv Respir Dis. 2015;9(1):3-15.

433. Oh GH, Yu JC, Choi KS, Joo EJ, Jeong SH. Simultaneous Comparison of Efficacy and Tolerability of Second-Generation Antipsychotics in Schizophrenia: Mixed-Treatment Comparison Analysis Based on Head-to-Head Trial Data. Psychiatry Investig. 2015;12(1):46-54.

434. Phan K, Xie A, Kumar N, Wong S, Medi C, La Meir M, et al. Comparing energy sources for surgical ablation of atrial fibrillation: a Bayesian network meta-analysis of randomized, controlled trials. Eur J Cardiothorac Surg. 2015;48(2):201-11.

435. Reinecke H, Weber C, Lange K, Simon M, Stein C, Sorgatz H. Analgesic efficacy of opioids in chronic pain: recent meta-analyses. Br J Pharmacol. 2015;172(2):324-33.

436. Schmid MK, Bachmann LM, Fas L, Kessels AG, Job OM, Thiel MA. Efficacy and adverse events of aflibercept, ranibizumab and bevacizumab in age-related macular degeneration: a trade-off analysis. Br J Ophthalmol. 2015;99(2):141-6.

437. Signorovitch JE, Betts KA, Yan YS, LeReun C, Sundaram M, Wu EQ, et al. Comparative efficacy of biological treatments for moderate-to-severe psoriasis: a network meta-analysis adjusting for cross-trial differences in reference arm response. Br J Dermatol. 2015;172(2):504-12.

438. Singh S, Garg SK, Pardi DS, Wang Z, Murad MH, Loftus EV, Jr. Comparative efficacy of pharmacologic interventions in preventing relapse of Crohn's disease after surgery: a systematic review and network meta-analysis. Gastroenterology. 2015;148(1):64-76.e2; quiz e14.

439. Sobieraj DM, Coleman CI, Pasupuleti V, Deshpande A, Kaw R, Hernandez AV. Comparative efficacy and safety of anticoagulants and aspirin for extended treatment of venous thromboembolism: A network meta-analysis. Thromb Res. 2015;135(5):888-96.

440. Stevens JW, Khunti K, Harvey R, Johnson M, Preston L, Woods HB, et al. Preventing the progression to type 2 diabetes mellitus in adults at high risk: a systematic review and network meta-analysis of lifestyle, pharmacological and surgical interventions. Diabetes Res Clin Pract. 2015;107(3):320-31.

441. Stynes G, Svedsater H, Wex J, Lettis S, Leather D, Castelnuovo E, et al. Once-daily fluticasone furoate/vilanterol 100/25 mcg versus twice daily combination therapies in COPD - mixed treatment comparisons of clinical efficacy. Respir Res. 2015;16:25.

442. Sun F, Chai S, Li L, Yu K, Yang Z, Wu S, et al. Effects of glucagon-like peptide-1 receptor agonists on weight loss in patients with type 2 diabetes: a systematic review and network meta-analysis. J Diabetes Res. 2015;2015:157201.

443. Sun F, Chai S, Yu K, Quan X, Yang Z, Wu S, et al. Gastrointestinal adverse events of glucagon-like peptide-1 receptor agonists in patients with type 2 diabetes: a systematic review and network meta-analysis. Diabetes Technol Ther. 2015;17(1):35-42.

444. Sun F, Wu S, Guo S, Yu K, Yang Z, Li L, et al. Effect of GLP-1 receptor agonists on waist circumference among type 2 diabetes patients: a systematic review and network meta-analysis. Endocrine. 2015;48(3):794-803.

445. Sun F, Wu S, Wang J, Guo S, Chai S, Yang Z, et al. Effect of glucagon-like peptide-1 receptor agonists on lipid profiles among type 2 diabetes: a systematic review and network meta-analysis. Clin Ther. 2015;37(1):225-41.e8.

446. Thakur D, Dickerson S, Kumar Bhutani M, Junor R. Impact of prolonged-release oxycodone/naloxone on outcomes affecting patients' daily functioning in comparison with extended-release tapentadol: a systematic review. Clin Ther. 2015;37(1):212-24.

447. van Walsem A, Pandhi S, Nixon RM, Guyot P, Karabis A, Moore RA. Relative benefit-risk comparing diclofenac to other traditional non-steroidal anti-inflammatory drugs and cyclooxygenase-2 inhibitors in patients with osteoarthritis or rheumatoid arthritis: a network meta-analysis. Arthritis Res Ther. 2015;17:66.

448. Wang C, Guo L, Chi C, Wang X, Guo L, Wang W, et al. Mechanical ventilation modes for respiratory distress syndrome in infants: a systematic review and network meta-analysis. Crit Care. 2015;19:108.

449. Wang L, Baser O, Kutikova L, Page JH, Barron R. The impact of primary prophylaxis with granulocyte colony-stimulating factors on febrile neutropenia during chemotherapy: a systematic review and meta-analysis of randomized controlled trials. Support Care Cancer. 2015;23(11):3131-40.

450. Wang Z, Qiao D, Lu Y, Curtis D, Wen X, Yao Y, et al. Systematic literature review and network meta-analysis comparing bone-targeted agents for the prevention of skeletal-related events in cancer patients with bone metastasis. Oncologist. 2015;20(4):440-9.

451. Wilhelmus KR. Antiviral treatment and other therapeutic interventions for herpes simplex virus epithelial keratitis. Cochrane Database Syst Rev. 2015;1:Cd002898.

452. Xiao J, Wu WX, Ye YY, Lin WJ, Wang L. A Network Meta-analysis of Randomized Controlled Trials Focusing on Different Allergic Rhinitis Medications. Am J Ther. 2015.

453. Yildiz A, Nikodem M, Vieta E, Correll CU, Baldessarini RJ. A network meta-analysis on comparative efficacy and all-cause discontinuation of antimanic treatments in acute bipolar mania. Psychol Med. 2015;45(2):299-317.

454. Zeng C, Li H, Yang T, Deng ZH, Yang Y, Zhang Y, et al. Electrical stimulation for pain relief in knee osteoarthritis: systematic review and network meta-analysis. Osteoarthritis Cartilage. 2015;23(2):189-202.

455. Zhu GQ, Shi KQ, Huang S, Huang GQ, Lin YQ, Zhou ZR, et al. Network meta-analysis of randomized controlled trials: efficacy and safety of UDCA-based therapies in primary biliary cirrhosis. Medicine (Baltimore). 2015;94(11):e609.

456. Zhu GQ, Shi KQ, Huang S, Wang LR, Lin YQ, Huang GQ, et al. Systematic review with network meta-analysis: the comparative effectiveness and safety of interventions in patients with overt hepatic encephalopathy. Aliment Pharmacol Ther. 2015;41(7):624-35.

# Appendix 4. Network meta-analysis terminology

| 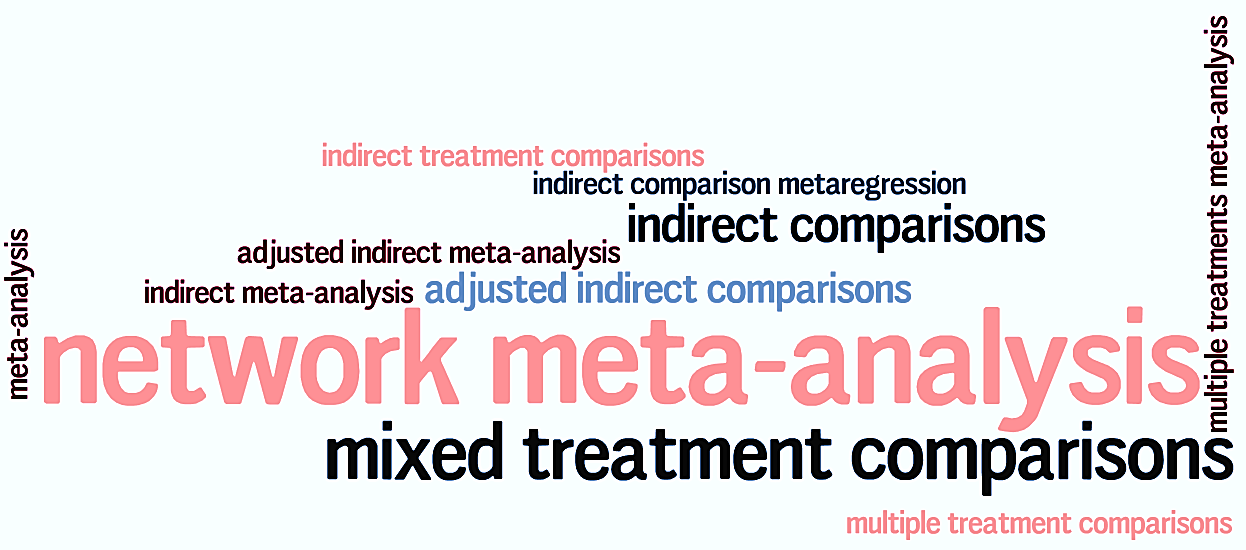 | |
| --- | --- |
| **NMA Terminology (n=456)** | *Count (%)* |
| Network meta-analysis | 213 (46.7%) |
| Mixed-treatment comparisons | 108 (23.7%) |
| Indirect comparisons | 56 (12.3%) |
| Adjusted indirect comparisons | 33 (7.2%) |
| Multiple-treatments meta-analysis | 16 (3.5%) |
| Multiple treatment comparisons | 9 (2.0%) |
| Indirect treatment comparisons | 9 (2.0%) |
| Meta-analysis | 8 (1.8%) |
| Indirect meta-analysis | 2 (0.4%) |
| Adjusted indirect meta-analysis | 1 (0.2%) |
| Indirect comparison meta-regression | 1 (0.2%) |

**Appendix 5. Methodology papers cited ≥ 10 times**

| **Most commonly cited papers (n=456)** | *Count (%)* |
| --- | --- |
| Lu & Ades 2004 [[1](#_ENREF_5_1)] | 137 (30.0%) |
| Caldwell 2005 [[2](#_ENREF_5_2)] | 76 (16.7%) |
| Bucher 1997 [[3](#_ENREF_5_3)] | 71 (15.6%) |
| Lumley 2002 [[4](#_ENREF_5_4)] | 37 (8.1%) |
| Salanti 2008a [[5](#_ENREF_5_5)] | 32 (7.0%) |
| Glenny 2005 [[6](#_ENREF_5_6)] | 30 (6.6%) |
| Song 2003 [[7](#_ENREF_5_7)] | 28 (6.1%) |
| Jansen 2011 [[8](#_ENREF_5_8)] | 25 (5.5%) |
| Ades 2006 [[9](#_ENREF_5_9)] | 23 (5.0%) |
| Dias 2011 [[10](#_ENREF_5_10)] | 21 (4.6%) |
| Dias 2010 [[11](#_ENREF_5_11)] | 20 (4.4%) |
| Jansen 2008 [[12](#_ENREF_5_12)] | 20 (4.4%) |
| Sutton 2008 [[13](#_ENREF_5_13)] | 13 (2.9%) |
| Hoaglin 2011 [[14](#_ENREF_5_14)] | 13 (2.9%) |
| Lu & Ades 2006 [[15](#_ENREF_5_15)] | 13 (2.9%) |
| Song 2009 [[16](#_ENREF_5_16)] | 12 (2.6%) |
| Lunn 2000 [[17](#_ENREF_5_17)] | 12 (2.6%) |
| Wells 2009 [[18](#_ENREF_5_18)] | 11 (2.4%) |
| Higgins & Whitehead 1996 [[19](#_ENREF_5_19)] | 11 (2.4%) |
| Salanti 2009 [[20](#_ENREF_5_20)] | 10 (2.2%) |
| Dias 2013b [[21](#_ENREF_5_21)] | 10 (2.2%) |
| Salanti 2011 [[22](#_ENREF_5_22)] | 10 (2.2%) |
| Not reported | 76 (16.7%) |

**References:**

1. Lu G, Ades AE. Combination of direct and indirect evidence in mixed treatment comparisons. Stat Med. 2004;23(20):3105-24.

2. Caldwell DM, Ades AE, Higgins JP. Simultaneous comparison of multiple treatments: combining direct and indirect evidence. BMJ. 2005;331(7521):897-900.

3. Bucher HC, Guyatt GH, Griffith LE, Walter SD. The results of direct and indirect treatment comparisons in meta-analysis of randomized controlled trials. J Clin Epidemiol. 1997;50(6):683-91.

4. Lumley T. Network meta-analysis for indirect treatment comparisons. Stat Med. 2002;21(16):2313-24.

5. Salanti G, Higgins JP, Ades AE, Ioannidis JP. Evaluation of networks of randomized trials. Stat Methods Med Res. 2008;17(3):279-301.

6. Glenny AM, Altman DG, Song F, Sakarovitch C, Deeks JJ, D'Amico R, et al. Indirect comparisons of competing interventions. Health Technol Assess. 2005;9(26):1-134, iii-iv.

7. Song F, Altman DG, Glenny AM, Deeks JJ. Validity of indirect comparison for estimating efficacy of competing interventions: empirical evidence from published meta-analyses. BMJ. 2003;326(7387):472.

8. Jansen JP. Network meta-analysis of survival data with fractional polynomials. BMC Med Res Methodol. 2011;11:61.

9. Ades AE, Sculpher M, Sutton A, Abrams K, Cooper N, Welton N, et al. Bayesian methods for evidence synthesis in cost-effectiveness analysis. Pharmacoeconomics. 2006;24(1):1-19.

10. Dias S, Welton N, Sutton A, Ades A. NICE DSU Technical Support Document 2: A generalised linear modelling framework for pairwise and network meta-analysis of randomised controlled trials: National Institute for Health and Clinical Excellence (NICE); 2011 [Available from: [www.nicedsu.org.uk](http://www.nicedsu.org.uk).

11. Dias S, Welton NJ, Caldwell DM, Ades AE. Checking consistency in mixed treatment comparison meta-analysis. Stat Med. 2010;29(7-8):932-44.

12. Jansen JP, Crawford B, Bergman G, Stam W. Bayesian meta-analysis of multiple treatment comparisons: an introduction to mixed treatment comparisons. Value Health. 2008;11(5):956-64.

13. Sutton A, Ades AE, Cooper N, Abrams K. Use of indirect and mixed treatment comparisons for technology assessment. Pharmacoeconomics. 2008;26(9):753-67.

14. Hoaglin DC, Hawkins N, Jansen JP, Scott DA, Itzler R, Cappelleri JC, et al. Conducting indirect-treatment-comparison and network-meta-analysis studies: report of the ISPOR Task Force on Indirect Treatment Comparisons Good Research Practices: part 2. Value Health. 2011;14(4):429-37.

15. Lu G, Ades AE. Assessing Evidence Inconsistency in Mixed Treatment Comparisons. Journal of the American Statistical Association. 2006;101(474):447-59.

16. Song F, Loke YK, Walsh T, Glenny AM, Eastwood AJ, Altman DG. Methodological problems in the use of indirect comparisons for evaluating healthcare interventions: survey of published systematic reviews. BMJ. 2009;338:b1147.

17. Lunn DJ, Thomas A, Best N, Spiegelhalter D. WinBUGS - A Bayesian modelling framework: Concepts, structure, and extensibility. Statistics and Computing. 2000;10(4):325-37.

18. Wells G, Sultan S, Chen L, Khan M, Coyle D. Indirect Evidence: Indirect Treatment Comparisons in Meta-Analysis. Ottawa: Canadian Agency for Drugs and Technologies in Health; 2009.

19. Higgins JP, Whitehead A. Borrowing strength from external trials in a meta-analysis. Stat Med. 1996;15(24):2733-49.

20. Salanti G, Marinho V, Higgins JP. A case study of multiple-treatments meta-analysis demonstrates that covariates should be considered. J Clin Epidemiol. 2009;62(8):857-64.

21. Dias S, Sutton AJ, Ades AE, Welton NJ. Evidence synthesis for decision making 2: a generalized linear modeling framework for pairwise and network meta-analysis of randomized controlled trials. Med Decis Making. 2013;33(5):607-17.

22. Salanti G, Ades AE, Ioannidis JP. Graphical methods and numerical summaries for presenting results from multiple-treatment meta-analysis: an overview and tutorial. J Clin Epidemiol. 2011;64(2):163-71.

**Appendix 6. Sources of grey literature searched**

| **Grey Literature Search Process** | | *Count (%)* |
| --- | --- | --- |
| **Grey literature searched (n=438)*** |  |  |
|  | Yes | 270 (61.6%) |
|  | No | 22 (5.0%) |
|  | Not reported | 146 (33.3%) |
| **Grey Literature Source (n=270)** |  |  |
|  | Conference/Dissertation Abstracts or Proceedings | 133 (49.3%) |
|  | Trial Registers | 131 (48.5%) |
|  | Relevant Organization and Agency Websites (e.g., WHO, CADTH) | 90 (33.3%) |
|  | Pharmaceutical Companies | 65 (24.1%) |
|  | Grey Literature Databases and Library Catalogues (e.g., Univeristy Catalogue, OpenSigle, LILACS) | 26 (9.6%) |
|  | Other sources (e.g., personal communication, textbook | 22 (8.1%) |
|  | Web Search Engines (e.g., Google, Google Scholar, Yahoo, Bing) | 19 (7.0%) |

** 18 NMAs did not provide details on the review method (e.g., literature search, study selection, data abstract and quality assessment)*

**Appendix 7. Quality appraisal tools used**

| **Quality Tools Used** |  | *Count (%)* |
| --- | --- | --- |
| **Quality Appraisal (n=438)*** | Reported | 345 (78.8%) |
|  | Not reported | 93 (21.2%) |
| **Quality Assessment Tool (n=345)** | Cochrane Risk of Bias tool | 130 (37.7%) |
|  | Jadad scale | 75 (21.7%) |
|  | Other | 68 (19.7%) |
|  | Author defined checklist | 36 (10.4%) |
|  | Cochrane ROB plus additional tool | 17 (4.9%) |
|  | No tools reported | 19 (5.5%) |

** NMAs without adequate reporting of review methods (e.g.,, literature search, study selection, data abstract and quality assessment) could not be included in AMSTAR assessment.*

# Appendix 8. AMSTAR assessment by items (n=438)*

** NMAs without adequate reporting of review methods (i.e., literature search, study selection, data abstract and quality assessment) could not be included in AMSTAR assessment.*

1. Was an 'a priori' design provided?
2. Was there duplicate study selection and data extraction?
3. Was a comprehensive literature search performed?
4. Was the status of publication (i.e. grey literature) used as an inclusion criterion?
5. Was a list of studies (included and excluded) provided?
6. Were the characteristics of the included studies provided?
7. Was the scientific quality of the included studies assessed and documented?
8. Was the scientific quality of the included studies used appropriately in formulating conclusions?
9. Were the methods used to combine the findings of studies appropriate?
10. Was the likelihood of publication bias assessed?
11. Was the conflict of interest included?

*Assessed based on guidance provided on:* [*https://amstar.ca/Amstar_Checklist.php*](https://amstar.ca/Amstar_Checklist.php)

# Appendix 9. Correlation between review duration and AMSTAR quality

# Appendix 10. AMSTAR quality overtime


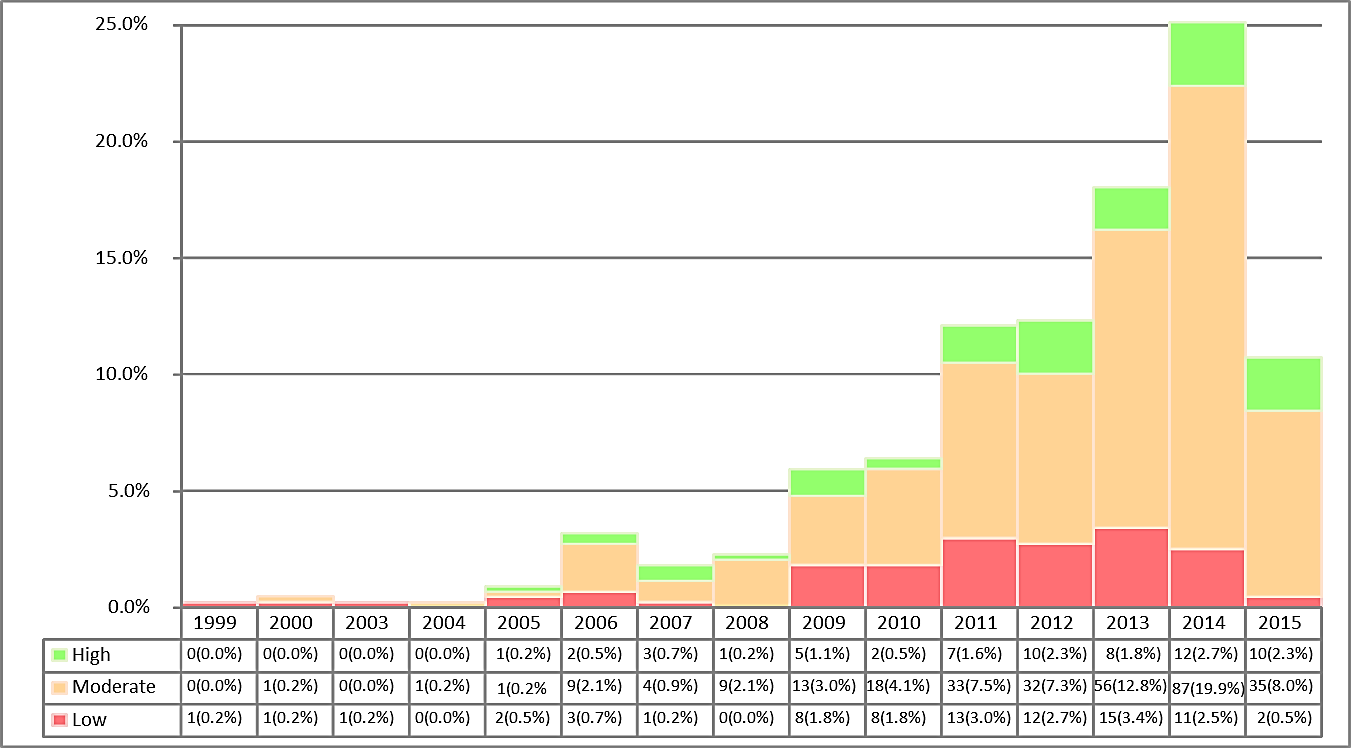


1. Nikolakopoulou A, Chaimani A, Veroniki AA, Vasiliadis HS, Schmid CH, Salanti G. Characteristics of networks of interventions: a description of a database of 186 published networks. PLoS One. 2014 Jan 22;9(1):e86754. [↑](#footnote-ref-1)
